# Analysis of the Microprocessor in Dictyostelium: The Role of RbdB, a dsRNA Binding Protein

**DOI:** 10.1371/journal.pgen.1006057

**Published:** 2016-06-06

**Authors:** Doreen Meier, Janis Kruse, Jann Buttlar, Michael Friedrich, Fides Zenk, Benjamin Boesler, Konrad U. Förstner, Christian Hammann, Wolfgang Nellen

**Affiliations:** 1 Department of Genetics, FB10, Kassel University, Kassel, Germany; 2 Ribogenetics Biochemistry Laboratory, Department of Life Science and Chemistry, Molecular Life Sciences Research Center, Jacobs University, Bremen, Germany; 3 Core Unit System Medicine, University of Würzburg, Würzburg, Germany; University of California Riverside, UNITED STATES

## Abstract

We identified the dsRNA binding protein RbdB as an essential component in miRNA processing in *Dictyostelium discoideum*. RbdB is a nuclear protein that accumulates, together with Dicer B, in nucleolar foci reminiscent of plant dicing bodies. Disruption of rbdB results in loss of miRNAs and accumulation of primary miRNAs. The phenotype can be rescued by ectopic expression of RbdB thus allowing for a detailed analysis of domain function. The lack of cytoplasmic dsRBD proteins involved in miRNA processing, suggests that both processing steps take place in the nucleus thus resembling the plant pathway. However, we also find features e.g. in the domain structure of Dicer which suggest similarities to animals. Reduction of miRNAs in the rbdB- strain and their increase in the Argonaute A knock out allowed the definition of new miRNAs one of which appears to belong to a new non-canonical class.

## Author Summary

miRNAs are essential regulators in eukaryotic cells and serve to control translation and stability of mRNAs. Processing of primary miRNA transcripts is carried out in two steps by evolutionary conserved machineries consisting mainly of double-strand specific RNases of the Dicer family and accessory double-strand RNA binding proteins (dsRBPs). Regulation occurs by effector proteins of the Argonaute family. While processing in plants is confined to the nucleus, the mechanisms is split into a nuclear and a cytoplasmic step in animals. By knock-out and complementation experiments, we identify RbdB in the amoebozoa *Dictyostelium* as the accessory dsRBP processing component for both steps. Fluorescence microscopy shows that RbdB co-localizes with the RNaseIII Dicer B in nucleolar foci suggesting mechanistic similarities to plants. Functional domain analysis of RbdB and the structure of Dicers, however, indicate similarities to animals. This places *Dictyostelium* at an evolutionary branch point between plants and animals. Deep sequencing reveals that the rbdB knock-out strain shows reduced accumulation of microRNAs. Comparison with the wild type and the miRNA overexpressing agnA knock-out strain, allowed for the identification of new miRNAs in *Dictyostelium* which may have escaped detection by other methods.

## Introduction

MicroRNAs (miRNAs) are an abundant class of regulatory RNAs that are encoded in the genome of eukaryotes. They are involved in many biological processes, e.g. in development, differentiation and metabolism [summarized in 1,2]. Key proteins of the RNAi-machinery are required for miRNA processing as well as for binding to cognate sequences in mRNA targets and subsequent gene silencing. At least one Argonaute-like protein, one Piwi-like protein, one Dicer and one RNA dependent RNA polymerase (RdRP) was suggested to have constituted the basic RNAi machinery in the last common ancestor of eukaryotes [3]. Research has focused on plants and animals which belong to the supergroups of archaeplastida and opisthokonta respectively [4]. Of special evolutionary interest is the supergroup of amoebozoa with the model organism *Dictyostelium discoideum* which branched off the tree of life after the plants but before the animal/fungi split [5]. *The D*. *discoideum* genome encodes five Argonaute proteins of the Piwi clade (AgnA to AgnE), two Dicer homologues (DrnA and DrnB) and three RdRPs (RrpA to RrpC). Previous experiments have shown that AgnA [6] and RrpC [7] are required for the production of siRNAs while DrnB is essential for miRNA processing [8,9]. DrnA is probably the nuclease that generates siRNAs but apparently, a knockout is lethal.

**Fig 1:**
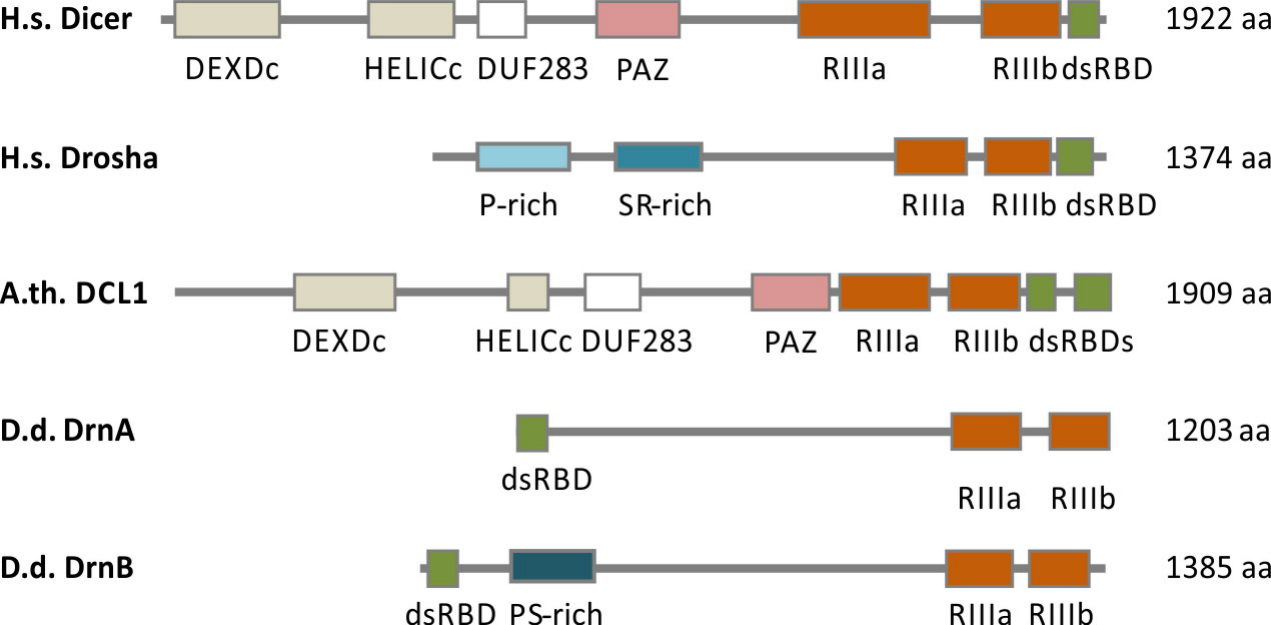
Domain architecture of RNase III enzymes. Schematic domain architecture of human Drosha and Dicer, DCL1 from *A*. *thaliana* (A.th.) and DrnA and DrnB from *D*. *discoideum* (D.d.) is shown. DsRBD (double stranded RNA binding domain), RIII (Ribonuclease III domain), HELICc (helicase superfamily c-terminal domain), DEXDc (Dead-like Helicases superfamily domain), PAZ (PAZ-domain), DUF283 (DUF283 domain). Human Drosha contains a region that is rich in prolines (P-rich site) as well as a region that is rich in serine and arginine (S/R-rich). DrnA and DrnB lack the Helicase domains, the DUF283 domain as well as the PAZ domain. DrnB exhibits a proline and serine rich (P/S-rich) region. Proteins and Domains are drawn to scale.

The structures of the *D*. *discoideum* RNase III proteins differ from those in animals or plants. Based on sequence similarities of the RNase III domains, DrnA and DrnB were classified as Dicer homologues, even though they lack a PAZ domain, the DUF283 domain and a helicase domain [3]. However, the latter is encoded in *D*. *discoideum* RdRPs instead [10]. Like animal Dicers, DrnA and DrnB encode one dsRBD instead of two as in plant Dicer homologues [3]. Surprisingly, the dsRBD is present at the N-terminus of DrnA and DrnB. Drosha like RNase III enzymes that are only present in animals are distinct in sequence from Dicer and consist of tandem RNase III domains and a C-terminal dsRBD [3]. Genes encoding proteins with these features are not present in the genome of *D*. *discoideum*. However, the domain architecture of DrnA and DrnB is more similar to that of Drosha-like enzymes than to Dicer homologues of animals and plants (Fig 1)

MiRNA transcripts (pri-miRNAs) are cleaved in two steps by RNase III nucleases: first, a hairpin-like precursor miRNA (pre-miRNA) is generated and then processed to a 21 nt long miRNA duplex. DsRNA binding domain proteins (dsRBPs) contribute miRNA processing [summarized in 11,12]. One strand of the miRNA duplex is stably incorporated into an Argonaute protein. Upon binding to perfect or partially complementary sequences in mRNA targets, the latter are endo- and exonucleolytically degraded or translationally repressed [summarized in 13]. Despite the similarities in miRNA processing of animals and plants, there are significant differences in the participating proteins and in compartmentalization.

In animals, pri-miRNAs are processed by the RNase III enzyme Drosha [14] in the nucleus which is assisted by the DiGeorge syndrome chromosomal region 8 (DGCR8) in humans [15–17] and Pasha in *D*. *melanogaster* and *C*. *elegans* [17,18]. Both are dsRNA binding proteins with two dsRBDs in a complex known as the microprocessor. Drosha and DGCR8 are nuclear proteins and partially co-localize at the nucleoli [19,20].

Pre-miRNAs are exported into the cytoplasm by Exportin-5 [21,22], where they are further processed to mature miRNAs by Dicer [23–25] in concert with dsRBPs: In *D*. *melanogaster* Dcr-1 interacts with Loquacious (Loqs), a protein with three dsRBDs that facilitates pre-miRNA formation [26,27]. Human Dicer is associated with the dsRNA binding protein TRBP or PACT and with Ago2 [28–30].

In plants, miRNAs processing depends on a nuclear complex consisting of the Dicer homologue DCL1 [31], the dsRBP HYL1 [32–34] and the zinc-finger protein Serrate (SE) [35,36]. DCL1 and HYL1 co-localize in discrete subnuclear structures which are known as dicing bodies (D-bodies) while Serrate only partially resides in these structures [37,38]. D-Bodies share different components with Cajal bodies [39]. HYL1 and DCL1 contain two dsRBDs with coordinated functions in miRNA processing: they mediate dsRNA binding, protein-protein interactions and help to target the proteins to D-bodies [32,37,40,41].

While the miRNA biogenesis pathway in animals and plants is quite well investigated [summarized in 42], much less is known in the Amoebozoa. With their unique position between animals and plants, the Amoebozoa and their siRNA machinery which cannot be unambiguously grouped to one or the other, provide an excellent model to understand RNAi evolution and the modular flexibility of this system.

Here we investigated the role of dsRBPs in *D*. *discoideum* miRNA generation in comparison to plants and animals.

## Results

### DsRBD containing proteins in *D*. *discoideum*

Since RNase III family members interact with dsRBD proteins to process miRNAs in plants and animals, we screened the genome of *D*. *discoideum* for proteins with homologous function. Using the InterPro algorithm, we were able to identify 10 annotated proteins with dsRBDs [43] (Table 1).

**Table 1:** dsRBD containing proteins in *D*. *discoideum* (AX4). *D*. *discoideum* proteins with dsRBDs were identified by an InterPro search [43]. Ribosomal and translation related proteins are shown in grey.

Accession	Protein name	*D*. *discoideum* database
P27685	40 S ribosomal protein S2	DDB_G0293742 [rps2]
Q54CA5	ribosomal protein	DDB_G0293090
Q54IC8	Class I peptide chain release factor	DDB_G0288835 [prfB]
Q54RE7	peptide chain release factor 1	DDB_G0283175 [prfA]
Q552X5	RNA binding domain protein A (RbdA)	DDB_G0275735
Q55E25	RNA binding domain protein B (RbdB)	DDB_G0269426
Q55FS1	Double stranded RNase B (DrnB)	DDB_G0268410 [drnB]
Q869Z1	DEAD/DEAH box helicase (Dhx9)	DDB_G0275313 [dhx9]
Q86L44	DEAD/DEAH box Helicase HelF	DDB_G0294407 [helF]
Q95ZG5	Double stranded RNase A (DrnA)	DDB_G0273051 [drnA-1]

**Fig 2:**
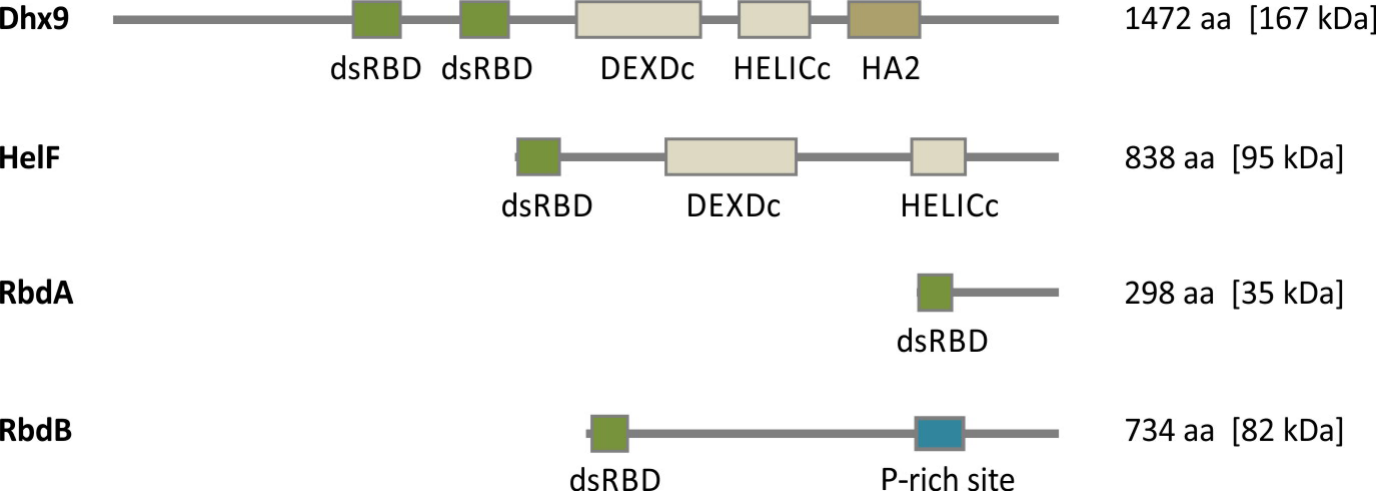
Schematic representation of dsRBD containing proteins in *D*. *discoideum*. **Dhx9** (1472 aa: dsRBD (1) [365–440], dsRBD (2) [532–607], DEXDc [715–903], HELICc [963–1069], HA2 [1132–1243]), **HelF** (837 aa: dsRBD [2–76], DEXDc [228–431], HELICc [608–687]), **RbdA** (297 aa: dsRBD [4–70]), **RbdB** (733 aa: dsRBD [9–75]), P-rich [510–584]. Numbers in brackets indicate the position of protein domains in the amino acid (aa) sequence predicted by SMART [45]. DsRBD (*double stranded* RNA *binding domain*), DEXDc (*Dead-like Helicases superfamily domain*), HA2 (*Helicase associated domain* 2), P-rich site (Proline rich site). Domains are drawn to scale.

We eliminated proteins which are involved in translation or ribosomal proteins and also excluded the RNase III proteins DrnA and DrnB. Dhx9 and HelF were interesting candidates, since they contain an RNA helicase domain which is absent in the *D*. *discoideum* dicer proteins. The knockout of *helF* had no effect on the miRNA processing in the amoeba [44] and the deletion of dhx9 is apparently lethal since no gene disruption could be obtained in several independent attempts and could not be analyzed. Two further uncharacterized proteins (DDB_G0275735 and DDB_G0269426) were identified and annotated *rbdA* and *rbdB* respectively. They contain a single dsRBD and no additional defined protein domains (see Fig 2).

### RbdB but not RbdA is involved in miRNA processing

We designed knockout strains where the promoter region and most of the dsRBD of *rbdA* and *rbdB* were deleted (S2 Table). Schematic presentation of the AX2 wild type (wt) and the mutant allele is shown in S1A Fig. At least two rbdA- and rbdB- strains each were obtained by independent transformations. Gene deletions were confirmed by PCR analysis and the BsR-cassette from the rbdB- strains was removed (S1B Fig). Deletion strains are referred to as rbdA- [2], rbdA- [3] and rbdB- [1] and rbdB- [2], respectively.

**Fig 3:**
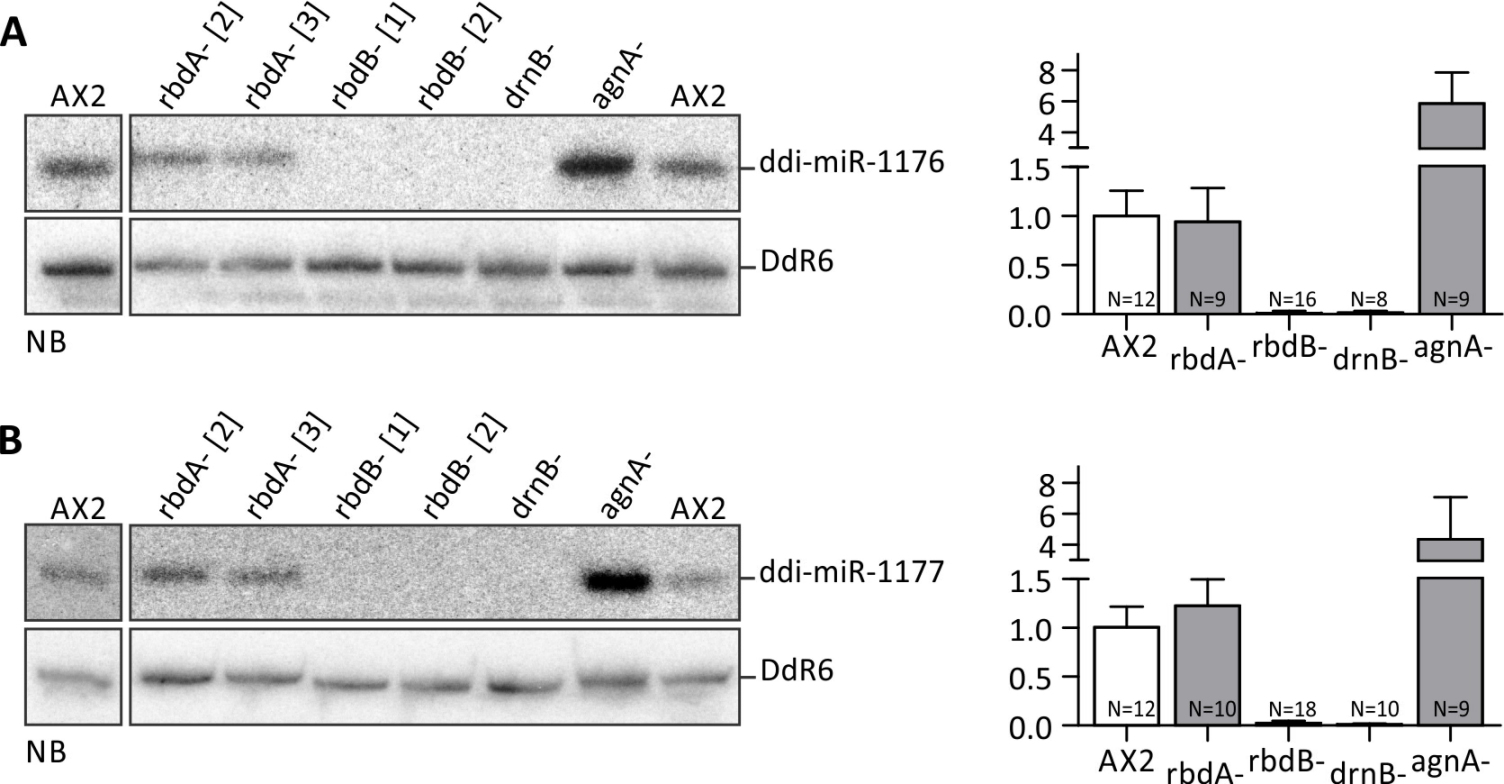
Mature miRNAs in rbdA and rbdB- strains. Expression levels of ddi-miR-1176 (A, left) and ddi-miR-1177 (B, left) were determined by Northern Blot analysis in the indicated knockout mutants. 12 μg total RNA were loaded per lane. Mature miRNAs were detected by ^32^P labeled oligonucleotides #2601 (α ddi-miR-1176) and #2602 (α ddi-miR-1177). To show equal loading, the membranes were rehybridized with a ^32^P labeled probe (#2654) against the snoRNA DdR6. The expression levels of ddi-miR-1176 (A, right) and ddi-miR-1177 (B, right) were quantified based on independent Northern Blots. Quantification of miRNA expression is given relative to DdR6 and was normalized to the AX2 wt (= 1). Error bars: mean with SD, paired t-test: ddi-miR-1176: AX2/rbdB- p < 0,0001 (***), AX2/drnB- p < 0,0001 (***), AX2/agnA- p < 0,0001 (***). Ddi-miR-1177: AX2/rbdB- p < 0,0001 (***), AX2/drnB- p < 0,0001 (***), AX2/agnA- p = 0,0026 (**). Number of n is given in the graph. For each mutant strain, at least two biological replicates were analyzed.

RbdA- and rbdB- strains were analyzed for molecular phenotypes in miRNA processing by Northern Blot using two known miRNAs. As a control, we analyzed RNA from the agnA- and the drnB- strains. The latter was known to lack endogenous miRNAs [9] (Fig 3).

While miRNA expression was similar to the wild type in the rbdA- strains, we could not detect any mature miRNAs in the rbdB- strains (Fig 3). They thus showed a similar molecular phenotype as the drnB- strain [9]. The same results were obtained with the original parent strains rbdB- [3] and rbdB- [5]. RbdB is thus necessary for proper miRNA processing. To our surprise, miRNA levels were strongly enriched in agnA- strains. Quantification revealed a 4-6-fold increase in the *agnA* mutant strain (Fig 3). The same molecular phenotype was recently observed upon deletion of the RdRP RrpC [8].

### Primary miRNA molecules accumulate in rbdB- strains

**Fig 4:**
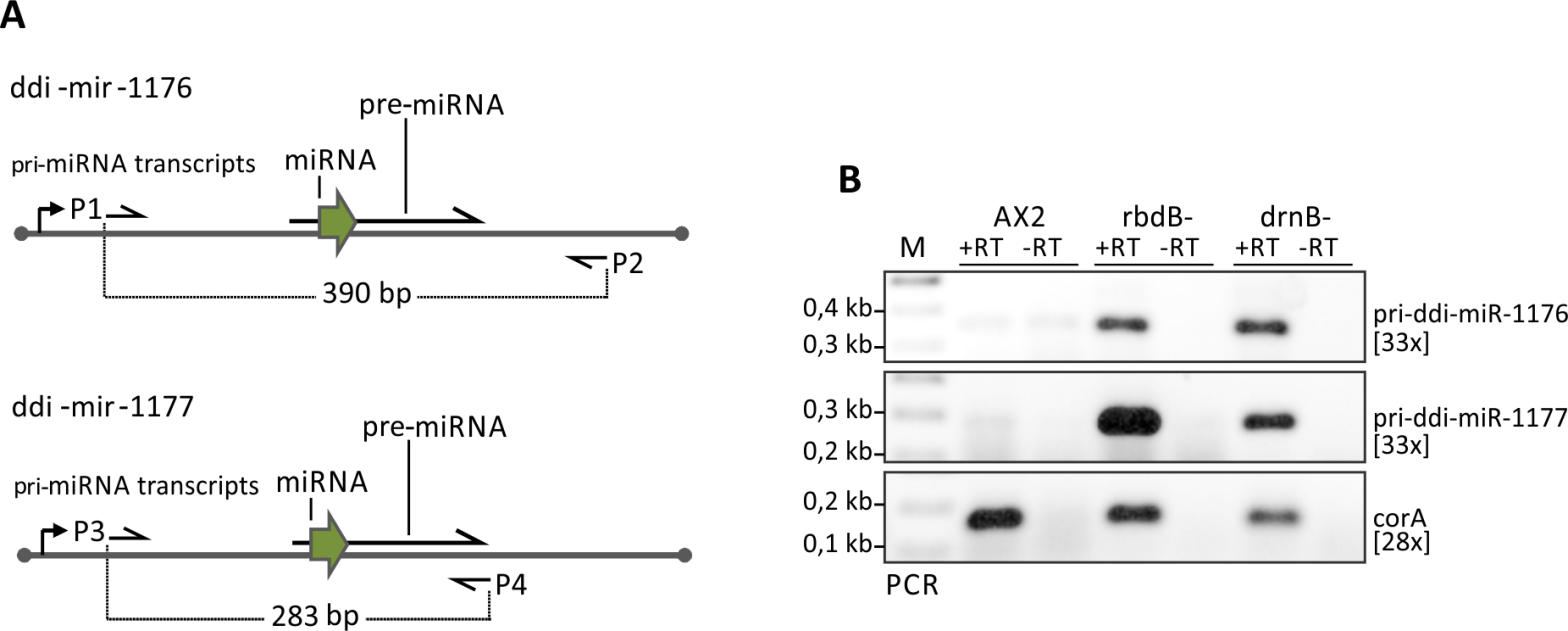
primary miRNAs accumulated in rbdB- and drnB- strains. A: Schematic representation of ddi-miR-1176 and ddi-miR-1177 transcripts and predicted pre-miRNA structures. Arrows indicate primers that were used to amplify primary miRNA transcripts. As a control, RT-PCR on *corA* mRNA was performed. B: Gene specific (reverse) primers were used to generate cDNA molecules: #1828 (*corA*), DM059 (pri-ddi-miR-1176), DM083 (pri-ddi-miR-1177). The primer sets P1/P2 (DM058/DM059), P2/P3 (DM082, DM083) and #1828/#1829 were used in the following PCR reaction to amplify pri-ddi-miR-1176 (390 bp), pri-ddi-miR-1177 (283 bp) and *corA* (200 bp) fragments respectively. The number in brackets indicates number of PCR-cycles. For unknown reasons, the minus RT control for pri-ddi-miR-1176 in the AX2 wild type always produced a weak signal.

To analyze if RbdB was involved in pri-miRNA or pre-miRNA processing, we performed RT-PCR analysis with primers outside the predicted pre-miRNA sequence. We show that primary miRNAs accumulated in rbdB- and in drnB- strains indicating that both proteins were involved in processing of pri-ddi-miR-1176 and pri-ddi-miR-1177 (Fig 4).

Since pri-miRNA processing occurs in the nuclei in plants as well as in animals, we further analyzed the subcellular localization of tagged RbdB.

### Subcellular localization of RbdB GFP fusions proteins

**Fig 5:**
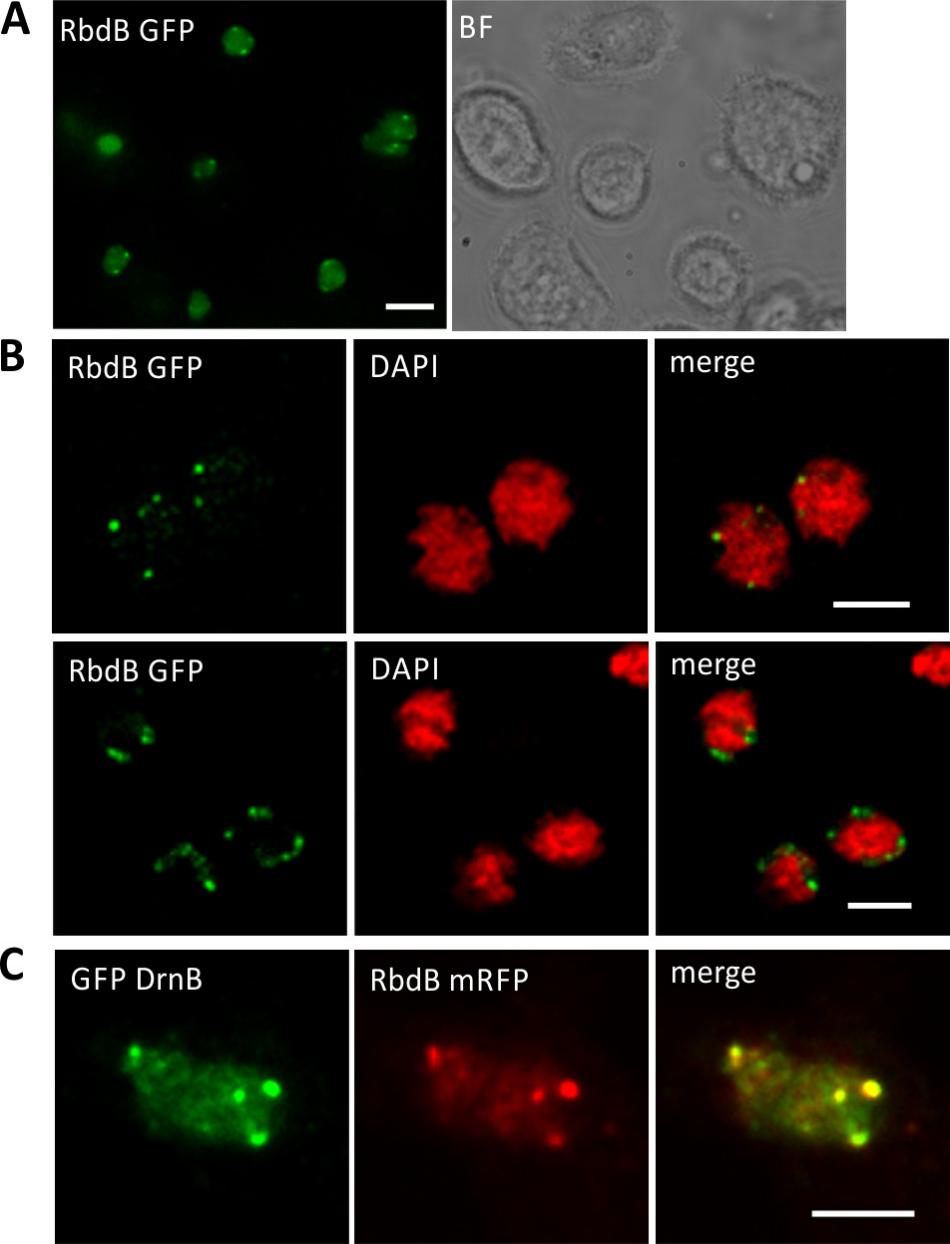
Subcellular localization of RbdB GFP and co-localization with DrnB. AX2 cells were transformed with the integrating plasmid pDneo2a RbdB GFP and subcellular localization was analyzed by fluorescence microscopy. A: Living cells were analyzed in low fluorescence axenic medium showing a diffuse distribution of the fusion proteins in the nucleoplasm and distinct foci at the periphery of the nuclei. Scale bar represents 5 μm. B: To better localize the subnuclear foci, cells were fixed with methanol and analyzed by an OptiGrid microscope (Leica DM 5500). Genomic DNA was stained by DAPI (red). The nucleoli showed no or only a very weak staining. Merging GFP (green) and DAPI (red) signals indicated that RbdB-GFP foci were enriched adjacent to areas with weak or no DAPI staining. Scale bar represents 2.5 μm. C: Co-localization of GFP DrnB and RbdB mRFP in nucleoli associated foci was monitored by fluorescence microscopy using methanol fixed cells. Shown is a single nucleus. Fusion proteins were expressed from extrachromosomally replicating plasmids. Scale bar represents 2.5 μm.

We expressed RbdB GFP fusions proteins in the wild type background and monitored fixed and living cells by fluorescence microscopy. RbdB GFP was found in the nuclei and was concentrated in discrete foci often associated with nucleoli (Fig 5). These structures were similar to plant D-bodies, in which the proteins DCL1, HYL1 and SE co-localize and interact [37]. The subnuclear distribution patterns of RbdB GFP were similar to those observed for DrnB fusion proteins [46] and in addition, both proteins were shown to co-localize (Fig 5C). We further showed that subcellular localization of DrnB and RbdB fusion proteins were not affected in rbdB- and drnB- strains, respectively (S2 Fig).

### Co-IP experiments confirm interaction of RbdB and DrnB

**Fig 6:**
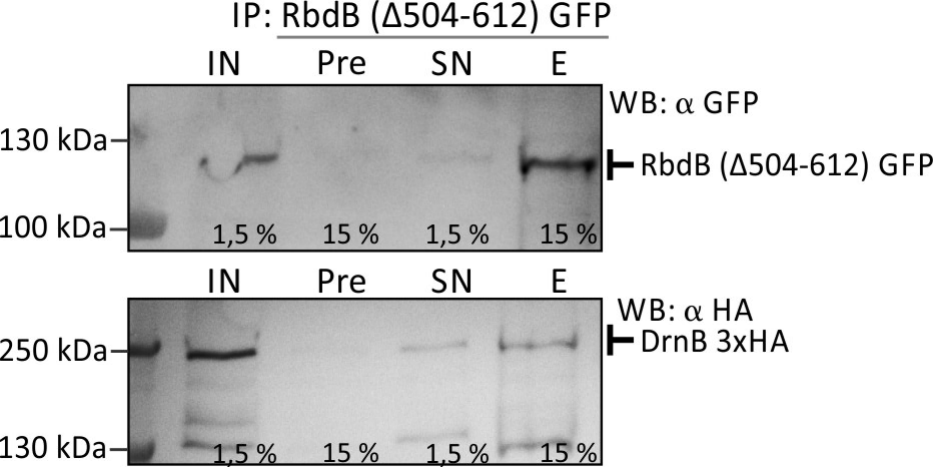
Co-IP of RbdB Δ 504–612 GFP and DrnB 3xHA. RbdB (Δ 504–612) GFP and DrnB 3xHA were expressed in the AX2 wt background. Co-immunoprecipitation of DrnB 3xHA by GFP tagged RbdB (Δ 504–612) was performed. Different samples (IN = input, Pre = preclear, SN = supernatant, E = elution) were analyzed by Western Blots. The fusion proteins were detected by specific α-GFP and α-3xHA antibodies. Numbers indicate the percent of input that was loaded on the SDS-gel. Control IPs were performed with strains expressing the nuclear localized HcpA GFP + DrnB 3xHA or RbdB (Δ504–612) GFP + HcpA 3xHA (S3 Fig).

To prove the interaction of RbdB and DrnB we co-expressed both proteins in the AX2 wt as GFP and 3xHA fusion proteins, respectively. For technical reasons, we used a truncated version of RbdB GFP, (RbdB Δ 504–612) that was shown to fully complement the mutant phenotype (see below). We used this protein as bait and could precipitate full length DrnB-3xHA (Fig 6).

### The Serrate (SE) ortholog in *D*. *discoideum* does not accumulate in nuclear foci

**Fig 7:**
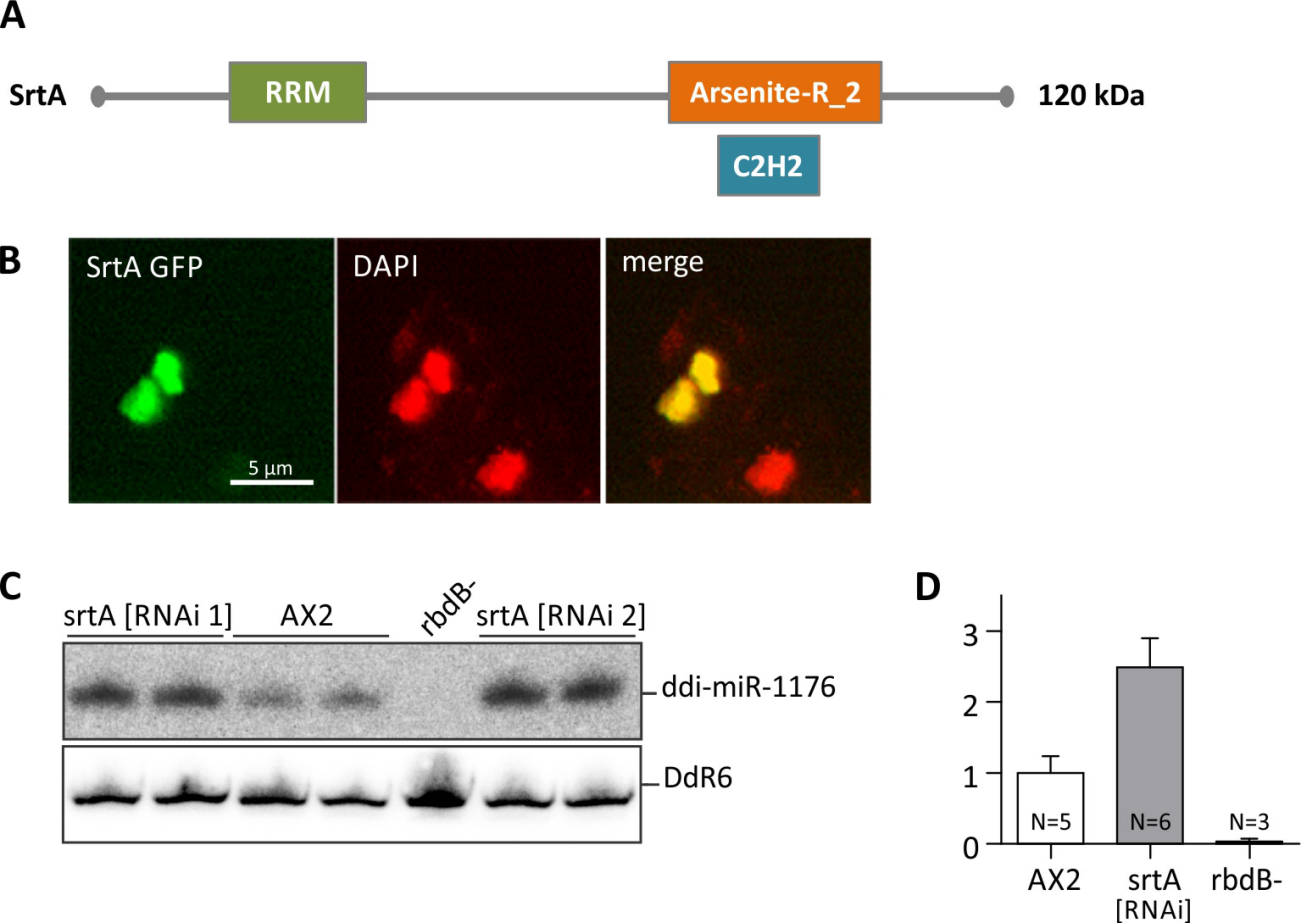
Subcellular localization of the Serrate ortholog (SrtA) in *D*. *discoideum*. A: Protein structure of the *D*. *discoideum* Serrate ortholog SrtA. RRM: RNA recognition motif domain, Arsenite-R_2: Arsenite-resistance protein 2, C2H2: Zinc finger domain [43]. B: AX2 cells expressing Srt GFP fusion proteins were fixed with methanol and analyzed by immunofluorescence. DNA was stained by DAPI (red). GFP (green) and DAPI signals were merged. C: ddi-miR-1176 miRNA processing was analyzed in AX2 and in srtA [RNAi 1] and srtA [RNAi 2] knockdown strains. 12 μg total RNA were loaded per lane. Mature ddi-miR-1176 was detected as described in Fig 3A. To show equal loading, the membrane was rehybridized with a probe directed against the snoRNA DdR6. D: The expression level of ddi-miR-1176 was quantified relative to DdR6 and normalized to the AX2 wt. Error bars: mean with SD, paired t-test: ddi-miR-1176: AX2/srtA [RNAi] p < 0, 0001 (***).

Serrate is involved in plant miRNA processing and is a component of D-bodies [35–37]. An ortholog (DDB_G0277375) in *D*. *discoideum* was predicted by InPranoid7 [47] and denominated *srtA* (Fig 7A).

SrtA was cloned and expressed as a GFP fusion in AX2 wt cells. We observed a diffuse localization in the nuclei and no nucleoli associated accumulation (Fig 7B). We then analyzed *srtA* mutant strains for miRNA processing. Since a knockout strain could not be generated, we constructed a srtA [RNAi] knockdown strain [48] and examined miRNA levels by Northern Blot. In contrast to expectations, ddi-miR-1176 was enriched (Fig 7C and 7D). A similar enrichment was observed for miRNA ddi-miR-7097 (S4A Fig) and for ddi-miR-1177. Analyses of a control knockdown strain verified that the effect was specific for srtA (S4B Fig). We confirmed the presence of *srtA* specific siRNAs by Northern Blot analysis (S4C Fig) and quantified knockdown efficiency by qPCR (S4C Fig) to an approx. 40% reduction in mRNA levels compared to AX2.

### Functional analysis of truncated RbdB variants *in vivo*

**Fig 8:**
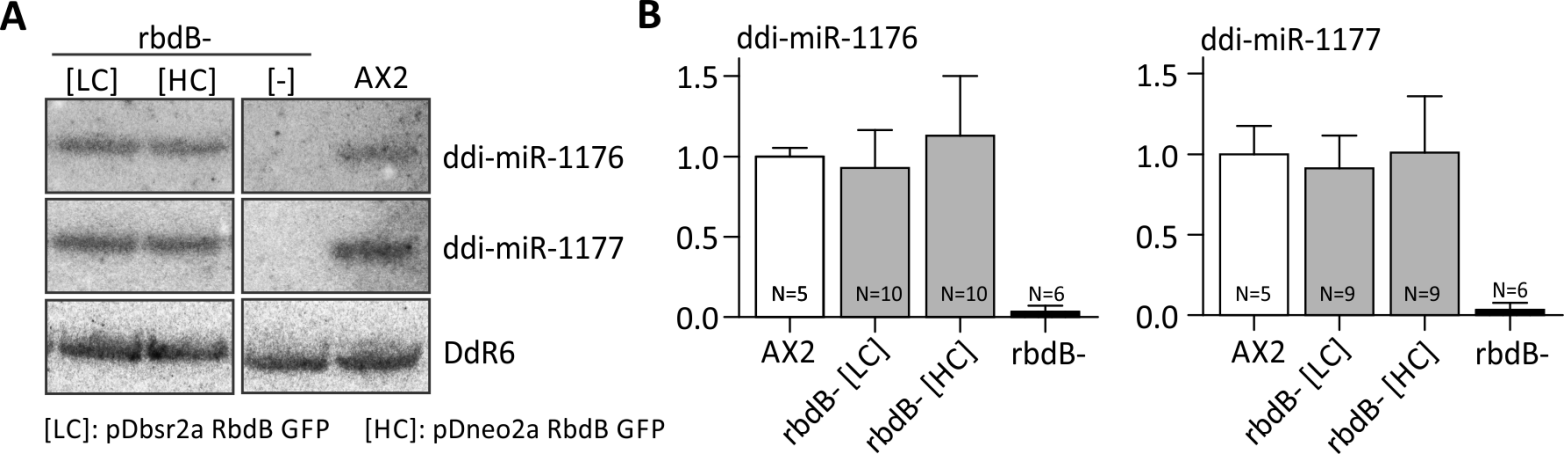
RbdB GFP fusion proteins fully complement the mutant phenotype. Independent rbdB- strains that were transformed with the plasmid pDbsr2a RbdB GFP [LC, low copy] or pDneo2a RbdB GFP [HC, high copy] were analyzed with respect to miRNA expression A: miRNA expression were analyzed by Northern Blot. 12 μg total RNA were loaded per lane. One clone with low RbdB GFP expression and one clone with high expression each are shown. As controls, we loaded RNA from the AX2 wt and from rbdB- strains. Mature miRNAs were detected by specific ^32^P labelled probes as described in Fig 3A. As a loading control, the membrane was finally rehybridized with a probe directed against the snoRNA Ddr6. B: miRNA signals from independent Northern Blots were quantified relative to the loading control and normalized to the AX2 wild type. According to paired t-test, no significant difference in expression levels was observed between the wild type and the different rescue strains.

We then tested if the rbdB- phenotype in miRNA processing could be complemented by ectopic expression of RbdB GFP fusion proteins. MiRNA levels (ddi-miR-1177 and ddi-miR-1176) were very similar to those in the AX2 wt, no matter if the protein was expressed from a high copy or from a low copy vector (Fig 8). Expression of the high copy and the low copy transgenes [49,50], as determined by qPCR for one biological replicate each, differed at least by a factor of 8 (S5A Fig) and GFP fusion protein expression was not even detectable in the low copy variant (S5B Fig). Overexpression of RbdB variants did not cause any mis-expression of miRNAs processing but always approached wild type expression levels.

**Fig 9:**
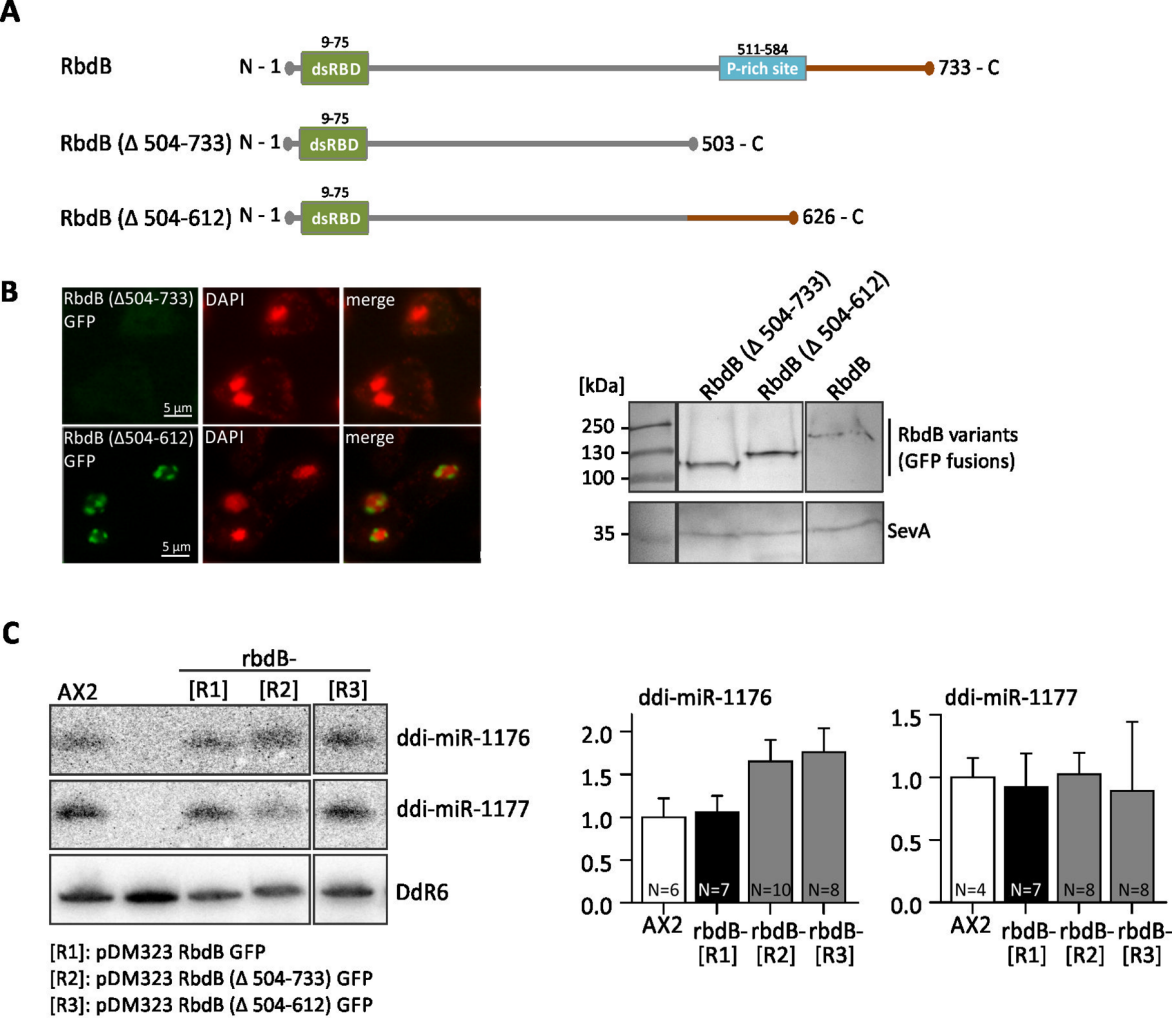
Analysis of truncated RbdB variants. A: Schematic representation of RbdB and truncated protein variants. In RbdB Δ504–733 GFP, 230 amino acids were deleted from the C-terminus. RbdB Δ504–612 GFP lacks the Prich-site. B: left: Both truncated RbdB-GFP versions were expressed in the knockout background and visualized by fluorescence microscopy. RbdB Δ504–612 GFP showed the same distribution as RbdB GFP. In contrast, RbdB Δ504–733 GFP was not detectably by fluorescence microscopy. right: Western Blot showing expression of RbdB Δ504–733 GFP (84 kDa), RbdB Δ504–612 (98 kDa) and of RbdB GFP (109 kDa). Note that all proteins run at higher molecular levels than calculated. SevA (40 kDa) is shown as loading control. C: left: Northern Blot analysis of rbdB- strains expressing RbdB GFP [R1], RbdB Δ504–733 GFP [R2], RbdB Δ504–612 GFP [R3] on miRNAs. 12 μg total RNA were loaded per lane. As a control, RNA from the AX2 wt and from an rbdB- strain was used. The mature miRNAs ddi-miR-1176 and ddi-miR-1177 were detected by ^32^P labelled probes as described in Fig 3A. Hybridisation to snoRNA DdR6 was used as a loading control. Right: miRNA signals (ddi-miR-1176 left, ddi-miR-1177 right) were quantified relative to DdR6 from different Northern Blots and normalized to the AX2 wt. R1-R3: rbdB- mutants were transformed with pDM323 RbdB Δ504–733 GFP (R1), pDM323 RbdB Δ504–612 GFP (R2) and with pDM323 RbdB GFP (R3). According to paired t-test, no significant difference was seen in miRNA accumulation between the wild type and the mutants.

RbdB contains a C-terminal region rich in Prolin and Threonin residues, reminiscent to the P-rich site in human Drosha. We generated two mutants: one where 230 C-terminal amino acids were deleted (RbdB Δ504–733) and one where only the P-rich site was deleted (RbdB Δ504–612) (Fig 9A). Both were introduced by extrachromosomal plasmids into rbdB- strains. Fluorescence microscopy of RbdB (Δ504–612) GFP fusion proteins showed the same localization as RbdB GFP. In contrast, RbdB (Δ504–733) GFP was not detectable by fluorescence microscopy (Fig 9B) but could be seen in Western Blots (Fig 9B). This was either due to misfolding of the GFP domain or to diffuse localization throughout the cell. Northern Blot analysis and quantification revealed that both deletion constructs complemented the mutant phenotype (Fig 9C).

Using cNLS Mapper [51] two overlapping bipartite nuclear localization signals (NLS1 aa 643–660, NLS2 aa 643–666) were predicted in the C-terminal region of RbdB. This region was still present in the truncated variant RbdB (Δ504–612) but not in RbdB (Δ504–733). In addition, we detected a putative nucleolar localization sequence (NoLS residues 693–713) using NoD [52]. We fused the coding regions for amino acids 643–713 (containing both signal sequences) and the NLS2 sequence alone to GFP for expression in the AX2 wt. The NLS2 sequence was sufficient to bring the reporter into the nucleus but no nucleoli associated foci could be observed in the presence of the NoLS signal sequence (S6 Fig).

### Identification of new miRNAs in *D*. *discoideum*

Since a strong decrease of known miRNAs has been observed in the rbdB- strain and an increase in the agnA- strain, we sequenced small RNAs from both strains and the wild type [6].

To identify putative miRNAs, we applied the following criteria. (1) Small RNAs should have a length between 20–24 nt. (2) The relative expression of putative miRNAs was at least 3-fold higher in agnA- strains compared to AX2 wt cells. (3) The relative expression of putative miRNAs was at least 3-fold lower in rbdB- strains than in AX2 wt cells. (4) The putative miRNA resides in a hairpin-like structure. (5) There is a putative corresponding miRNA-5p or -3p sequence. (6) miRNA-5p or miRNA-3p are detectable by Northern Blot. We considered a miRNA as validated when at least four of these criteria were met. In addition to some of the known miRNAs, including those of a recent study [8], we detected 4 new species (Table 2), three of which fulfilled the criteria for canonical miRNAs. Since the read number per strain was relatively low, this analysis is by far not complete but only demonstrates a proof of principle. S3 Table shows absolute read counts of miRNAs in the different strains. S7 Fig shows relative expression of the miRNA candidates. Notably, the miRNA-like non-canonical small RNA (miRNA-like_D4) matched four positions (S4 Table) with two very close to the telomeres. The 22 nt long RNA showed an elevated expression level in the agnA- strain based on the deep sequencing studies but no significant differences between the AX2 wt and the rbdB- strain. Additionally, the flanking sequences did not fold into a canonical hairpin-like structure and did thus not fulfill the official criteria of a canonical miRNA [53].

**Table 2:** Identification of new miRNAs. Canonical and non-canonical miRNAs. nd = not detectable in RNAseq. The miRNA ddi-mir-7097 was identified by Avesson et al. 2012 but only with very low read counts and lack of the miRNA-3p.The exact 5’ and 3’end may deviate by a few nucleotides. Relative expression levels of miRNAs in different strains are shown based on RNA-sequencing data. Reads were extracted from the genomic positions given in S4 Table (+/- 5 nucleotides) and divided by the total read number. Data were then normalized for AX2.

*miRNA	sequence [nt]	criteria	relative expression		
*miRNA			AX2	rbdB-	agnA-
**known miRNAs**					
ddi-miR-1176-5p [9]	CCAAUUUUUAUCAAGGAAAGC	1,2,3,4,5,6	16,00	3,30	1341,16
ddi-miR-1176-3p	CUUCCUUGACAAAAAUUGCCC		1,00	0,00	1,11
ddi-miR-1177-5p [9]	CCAGUUAGGGUUUAAUGGUUC	1,2,3,4,5,6	3,00	0,00	36,52
ddi-miR-1177-3p	ACCGUUGAGCCCUUUCUGAUU		7,00	0,00	167,09
**identified canonical miRNAs**					
miRNA_can_D1-5p	UCUUUCUCUAAUUUCAUUUAUU	1,2,3,4,5,6	54,00	0,00	588,69
miRNA_can_D1-3p	AAAUGAAAUUAGAGAAAGGGAU		8,00	1,65	79,67
miRNA_can_D2-5p	UUCUCGACAGACAUAGCAUUGG	1,2,3,4,5,6 (in rrpC- and agnA-)	14,00	1,65	915,13
miRNA_can_D2-3p	AAUGCUUAGAUGUAGAGGAAAC		0,00	0,82	9,96

ddi-mir-7097-5p [8]	UCUCUACUAGUGCCGAAAUCA	1,2,3,4,5,6	66,00	12,36	2599,33
ddi-mir-7097-3p	UUUGGCAGAAGUAGAGACGAA		10,00	5,77	590,91
miRNA_can_D3-3p	AUUAAUUUCGGCAGCCAUAUU	1,2,3,4,5,6	68,00	3,30	185,90
miRNA_can_D3-5p	UAUGGCUGCUGAAAUUAAUGUGG		47,00	26,37	369,59
**non-canonical miRNA**					
miRNA-like_D4	UCGAACUAGUCCAAUCUUUAAU	1,2,3 (based on NB), 6, no hairpin	12,00	754,68	24,72

**Fig 10:**
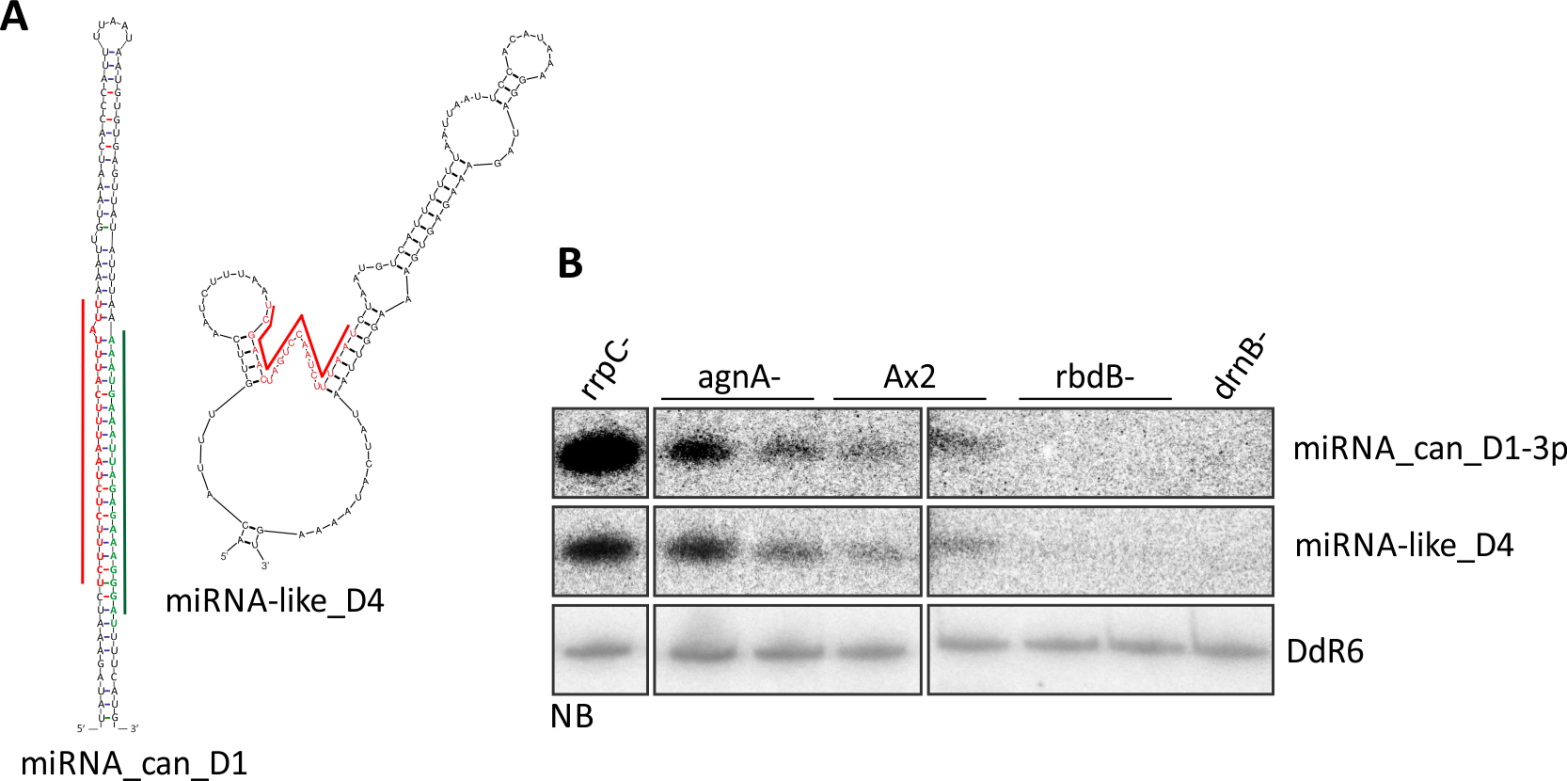
Validation of miRNA candidates. A: Newly identified miRNAs were examined for putative secondary structures by M-fold (Zuker et al., 2003). MiRNA_can_D1 and the corresponding miRNA-3p fold into a typical hairpin structure, whereas miRNA-like D4 did not. Folding is shown for one locus on chromosome 6, coordinates 3669–3690. Red: miRNA-5p, green: miRNA-3p. B: 12 μg of total RNA from the strains indicated were separated on an 11% PAA gel and transferred to a nylon membrane and putative miRNAs-3p or miRNAs-5p were detected with ^32^P labelled oligonucleotides. For miRNA_can_D1, only the miRNA-3p was detected by Northern Blot.

As an example, validation (criteria 5 and 6) is shown for the canonical miRNA_can_D1 and for the non-cononical miRNA-like D4 (Fig 10).

In Northern Blots the small RNA behaved like a canonical miRNA in terms of expression patterns in the RNAi-mutant strains. It may constitute a new class of miRNAs which is generated by cleavage in the adjacent stem-loop structures. This is somewhat supported by the observation that no corresponding miRNA-5p or -3p was found. Another non-canonical miRNA (miRNA-like _D3) was located in the intron of the DNA transposon thug-S [54] and is thus encoded several times in the genome (S4 Table). Thug-S derived miRNAs were already published by Avesson et al. 2012. However, the great majority of these RNAs were found in developed cells (16 hours and 24 hours RNA libraries) [8]. We were able to detect thug-S derived miRNAs by Northern Blots in vegetative cells although very weakly (S8 Fig). Northern Blot analysis and folding analysis for the remaining small RNAs are also shown in S8 Fig. The canonical and miRNA-like miRNAs are listed in Table 2.

## Discussion

### miRNA processing in *Dictyostelium discoideum*

We have shown that the dsRNA binding protein RbdB is a necessary component for miRNA processing in *D*. *discoideum*. It interacts with the nuclear protein DrnB, which has previously been shown to be required for miRNA accumulation [8,9]. Disruption of the rbdB gene did not result in an obvious mutant phenotype in growth or development under laboratory conditions but in a molecular phenotype in that previously identified miRNAs were almost entirely lost. In contrast, the disruption of the closely related rbdA gene had no such effect.

In agreement with the subcellular localization of RbdB, pri-miRNAs were found to be enriched in rbdB- strains and the same was true for drnB- cells. Both proteins are thus at least required to convert pri-miRNAs to pre-miRNAs. Since rbdA- and helF- strains show normal miRNA accumulation, no other cytoplasmic dsRBP appears to be involved in the accumulation of mature miRNAs. We can, however, not rule out the unusual case that some other non-dsRBD proteins adopted this function. The knockout of DrnA is apparently lethal since no clones could be obtained in multiple independent attempts.

In *Arabidopsis*, protein interactions in D-bodies are mediated by the second dsRBD of DCL1 and probably by the second dsRBD of HYL1 [32,41]. Drosha requires DGCR8 and its two encoded dsRBDs for accurate and efficient pri-miRNA processing [55], [16].

The zinc-finger protein Serrate is another component of the microprocessor-like complex in plants and contributes to miRNA processing. A knockdown of the *D*. *discoideum* homologue SrtA resulted in an unexpected enrichment of miRNAs. This suggests that SrtA plays a direct or indirect role in miRNA processing which is different from that in plants. However, due to the relatively low knockdown efficiency of 40% mRNA reduction, we can only speculate that Serrate in *D*. *discoideum* has adopted the role of a negative regulator of miRNA processing and may compete with the microprocessor complex of DrnB and RbdB.

### Subcellular Localization of the microprocessor-complex

DrnB localizes in distinct subnuclear compartments which can often be found in close association with nucleoli [46]. RbdB GFP fusions had a similar distribution and both proteins co-localize in these structures. This is reminiscent of plant Dicing Bodies, where HYL1 and DCL1 co-localize.

The microprocessor in *D*. *discoideum* appears to be strictly confined to the periphery of the nucleoli. A canonical NLS was found in RbdB and we show that it was necessary and sufficient for nuclear import but not for localization in D-bodies. Interestingly, this precise localization seems not to be necessary for function since several mutant constructs diffusely localize in the nuclei or even in the cytoplasm but still rescue the phenotype in rbdB- strains. We assume that small but sufficient amounts of the protein, undetected by fluorescence microscopy, are transported to the localized microprocessor. This occurs apparently not by co-import of DrnB and RbdB since both proteins localize correctly even if the other one is knocked out. Neither in plants nor in *D*. *discoideum* it is, however, known if D-bodies are the location of miRNA processing, they may also be storage particles for the microprocessor and be dispensable for miRNA generation.

### MiRNA–Definition in *D*. *discoideum*

We have previously shown that the argonaute protein AgnA is required for siRNA production [6]. Here we demonstrate a direct or indirect negative effect of AgnA on miRNA accumulation since all examined miRNAs display significantly higher levels in agnA- cells.

By the criteria of overexpression in agnA- cells and underexpression in rbdB- cells we have identified, as a proof of principle, 4 new miRNAs and verified their existence by Northern blot. Surprisingly, one of them could not be folded into a hairpin by Mfold. We termed this a miRNA-like small RNA. Consistently, we find no evidence for a corresponding miRNA5p or -3p at this locus. A second miRNA with essentially the same features was detected by deep sequencing but was barely detectable in Northern Blots. Such miRNA-like small RNAs would probably have escaped most prediction tools. It will require further investigations to determine if these molecules are derived from a new class of precursors that may be processed by alternative pathways. One may speculate that the predicted hairpin structures adjacent to the unpaired miRNA region may serve as unconventional processing sites for DrnB.

Taken together, our data suggest a hybrid mechanism from plants and animals in *D*. *discoideum*: the RNase III enzymes are Dicer-like in sequence but more similar to animal Droshas in domain structure. There is a nuclear Serrate homolog in *D*. *discoideum* that is directly or indirectly involved in miRNA processing but it appears to have adopted a different function from that in plants. A similar functional role was found for AgnA since deletion of either gene resulted in an accumulation of miRNAs. Probably, both miRNA processing steps are carried out in the nucleus as in plants. This is suggested by the lack of suitable cytoplasmic dsRBD candidates that are usually required for generating mature miRNAs and by the presence of D-bodies in the nucleolar periphery. Furthermore, our data emphasize the modular character and the flexibility of the RNAi machinery: functional domains have been exchanged between proteins in the microprocessor during evolution and can still be shuttled by molecular methods without affecting the functionality of the complex.

## Materials and Methods

### Cell growth and transformation

All *D*. *discoideum* strains were grown axenically in HL5+ medium (Formedia) supplemented with Blasticidine S and/or Geneticin at concentrations of 10 μg/mL when required. Transformation into the axenic strain AX2 or derivates was done by electroporation as described previously [56]. When integrating plasmids or knockout constructs were transformed, cells were subcloned in order to isolate single colonies. Clones were considered independent when they were derived from different transformations. After transformation with extrachromosomal vectors, cell populations were used for further analysis.

#### rbdA and rbdB knockout strains

The left (-999 to -552) and the right arm (+157 to +749) of the *rbdA* gene (accession number: DDB_G0275735) knockout construct were amplified with the primers BB100/BB101 and BB102/BB103 respectively. Using the Stargate Transfer Reagent Set (IBA) the arms were shuttled in the pKOSG-IBA-dicty1 destination vector (IBA) where they flanked a BsR-cassette [57]. The left arm (-883 to -503) and the right arm (+362 to +1106) of the *rbdB* (accession number: DDB_G0269426) knockout construct were amplified with the primers BB104/BB105 and BB106/BB107. The knockout vector was generated as described for the *rbdA* knockout plasmid. Gene disruption fragments were cut out from the vector backbone with PstI and transformed into AX2 wt cells. Mutants were analyzed by PCR. The BsR cassette from the rbdB- strain was removed by transient expression of Cre-recombinase [58].

#### RbdB overexpression strains

The gene for *rbdB* (accession number: DDB_G0269426) was amplified with the primers DM033 and DM034 from genomic DNA. It was ligated into the integrating plasmids pDneo2a GFP and pDbsr2a GFP [46] via the PstI site and thus fused to a C-terminal GFP-tag. These plasmids were then transformed into AX2 wt or in rbdB- strains. For rescue experiments, we transformed the integrating plasmids pDneo2a RbdB GFP and pDbsr2a RbdB GFP twice in rbdB- [1] and rbdB- [2] strains. After subcloning, we isolated two different clones from each transformation.

In order to express RbdB from extrachromosomal vectors, the gene was amplified with the primers DM066 and DM053 and ligated into the pJET1.2/*blunt* cloning vector. The gene was cut out with BclI and SpeI and ligated into the vector pDM323 [59] which was linearized with BglII and SpeI and that contained a GFP-gene downstream of the MCS. The sequence of *rbdB* Δ504–733 was amplified from genomic DNA with the primers DM066 and JB002. Moreover, a 5’ BclI and a 3’ SpeI site were added during the PCR-reaction. The fragment was cloned in the pDM323 expression vector via BglII/SpeI and thus fused to a C-terminal GFP-tag. In this version, the complete C-terminal part of the gene was missing. The sequence of *rbdB* Δ504–612 was cloned in two steps: The N-terminal part was amplified with the primers DM066 and DM065 and an N-terminal BclI and a C-terminal BglII site were added. The PCR-fragment that did not cover the P-rich site and was cloned into the pGEM-T Easy vector (Promega). The C-terminal part of the rbdB gene was cut out from a vector that contained a full length version of the rbdB gene flanked by BclI and SpeI (pJET1.2/blunt BclI rbdB SpeI) using BglII. One BglII site was present in the gene for rbdB itself (downstream of the P-rich site) and another one in the MCS of the cloning vector downstream of the SpeI site. This fragment was cloned adjacent to the N-terminal rbdB fragment in the context of the pGEM-T Easy vector. Thus, sequences encoding the amino acids Δ504–612 were deleted. It should be noted that not only the P-rich site which comprises the amino acids 511–584 was cut out but also a few adjacent nucleotides. The complete sequence encoding RbdB Δ504–612 was cut out from the cloning vector by BclI and SpeI and ligated into the pDM323 expression vector that was linearized with BglII and SpeI.

All extrachromosomal expression vectors were transformed in rbdB- or in the AX2 wt strains. Different populations from independent transformation events were analyzed.

#### GFP DrnB/RbdB mRFP overexpression strain

The genomic sequence of drnB was amplified and cloned into the integrating vector pDneo2a GFP previously [46]. Full length DrnB was re-amplified by PCR for cloning into pDM317 [59]. The BclI and SpeI flanked gene for rbdB was ligated into pDM326 [59] featuring a Blasticidin S resistance marker. Plasmids were transformed in the AX2 background.

#### rbdB- DrnB GFP and drnB- RbdB mRFP strains

The gene for drnB was amplified from the vector pDneo2a GFP [46] using primers FZ005 and FZ008 and ligated into the pDM323 [59] vector. The plasmid was then transformed in the rbdB- strain. The plasmid pDM326 RbdB mRFP featuring a Blasticidin S resistance gene was transformed in the drnB- strain, respectively.

#### Serrate (srtA) knockdown strain: AX2 srtA [RNAi]

The knockdown plasmid for the *Dictyostelium discoideum* Serrate (SE) orthologue (DDB_G0277375) was cloned as follows: the primers DM200 and DM201 were used to amplify the trigger from cDNA (+613 to 1436 (coding sequence)). The fragment was inserted in the pJET1.2/*blunt* cloning vector. Finally, the trigger sequence was cut out with BglII and SpeI and inserted in the DIRS-1 based knockdown vector pDM304 lITR MCS rITR [48] that was linearized with the same restriction enzymes. SrtA siRNAs were monitored by Northern Blot analysis. We do, however, not know the level of protein reduction since no antibody against SrtA is available.

#### Serrate (srtA) overexpression strains

Serrate (*srtA*) was expressed from extrachromosomal vectors in the AX2 wt background. The sequence (DDB_G0277375) was amplified in two parts form genomic DNA. The N-terminal part was amplified with the primers DM196 and DM197. By primer DM196, a BamHI site was added. Primer DM197 flanked the endogenous KpnI site. Using primers DM198 and DM199, the C-terminal SE part was amplified. The reverse primer added a SpeI site. Both fragments were cloned independently in the pGEM-T Easy cloning vector (Promega) in forward orientation. The vector containing the C-terminal part of the gene was cut with KpnI and SpeI (in the MCS). The N-terminal part was cut out of the cloning vector with the same enzymes and ligated into the linearized vector. The resulting full length SE gene was cut out by BamHI and SpeI and then ligated into the extrachromosomal expression vectors pDM317 and pDM323 [59], that were linearized by BglII and SpeI. Both plasmids were transformed twice in the AX2 wt resulting in srtA [RNAi 1] and srtA [RNAi 2].

#### RbdB Δ 504–612 GFP + DrnB 3xHA overexpression strains

Both fusion proteins were expressed from the same plasmid. The gene for drnB was amplified from the plasmid pDneo2a-GFP-DrnB [46] with the primers FZ005 and FZ008 and then ligated in the pJET1.2/*blunt* vector. The gene was cut out with BglII and SpeI and ligated into the plasmid pDM344 3xHA (C-term) that had been linearized with the same restriction enzymes. The shuttle vector pDM344 3xHA (C-term) was generated as follows: a 3xHA tag was amplified by recursive PCR, using the primers #3169 and #3170. An N-terminal SpeI and a C-terminal XbaI site were added via the primers. The fragment was ligated into the pJET1.2/*blunt* vector. The tag was cut with SpeI and XbaI and inserted in the pDM344 vector [59] that was linearized with SpeI. By this, the multiple cloning site (BglII/SpeI) was maintained. The DrnB 3xHA expression cassette was cut out from the shuttle vector with NgoMIV and ligated into the vector pDM323 RbdB Δ 504–612 GFP that had been linearized with the same restriction enzyme. The expression plasmid encoding RbdB (Δ 504–612) GFP and DrnB 3xHA was transformed in the AX2 wild type strain.

#### GFP + DrnB 3xHA overexpression strains

The expression cassette of DrnB 3xHA was cut out from the shuttle vector pDM344 3xHA (C-term) with NgoMIV and ligated into the pDM317 vector that had been linearized with the same restriction enzyme. The expression plasmid encoding GFP and DrnB 3xHA was transformed in the AX2 wild type strain.

#### GFP HcpA + DrnB 3xHA and RbdB (Δ504–612) GFP + HcpA 3xHA overexpression strains

The gene for *hcpA* was amplified from the plasmid pDd-HcpA-GFP [60] with the primers #2971/#2972. By PCR, an N-terminal BamHI and a C-terminal SpeI site were added. Via these restriction sites the *hcpA* gene was ligated into the pDM317 expression vector and into the pDM344 shuttle vector that contained a 3xHA tag downstream of the MCS. The expression cassette for drnB 3xHA was cut out from the shuttle vector pDM344 BglII DrnB SpeI 3xHA with NgoMIV. The isolated fragment was inserted into the pDM317 HcpA expression vector via the single NgoMIV site resulting in the plasmid pDM317 GFP HcpA + DrnB 3xHA. In order to clone the plasmid pDM323 RbdB (Δ504–612) GFP + HcpA 3xHA the expression cassette for HcpA 3xHA was cut out from the pDM344 shuttle vector via NgoMIV. The fragment was then ligated in the plasmid pDM323 RbdB Δ504–612 that has been linearized with the same restriction enzyme before. Both plasmids were transformed in the AX2 wild type strain.

#### Verification of predicted NLS or NoLS signal sequences

The residues 643–713 of the rbdB-gene containing the predicted NLS and NoLS sequences were amplified with the primers DM167 and DM172 and ligated in the pDM317 vector via the SpeI and BglII sites. Thus, the residues 643–713 of rbdB were fused C-terminal to the GFP-tag. The NLS2 sequence alone was generated by recursive PCR using the overlapping pimers DM208 and DM209. The NLS2 sequence was fused C-terminal to the GFP tag in the pDM317 vector via BglII and SpeI as well.

### Oligonucleotides

DNA oligonucleotides (Invitrogen) used in this study are listed in S1 Table.

### Analysis of RNA by Northern Blot

Isolation of total RNA from *D*. *discoideum* and Northern Blot analysis of small RNAs were performed as described previously [6]. Blots were probed with 5’ ^32^P labeled DNA oligonucleotides that are listed in S1 Table.

### qRT-PCR analysis

qRT-PCR analysis were performed as described elsewhere [6].

### Deep sequencing of small RNAs

Illumina sequencing of small RNAs (<400 nt) from the AX2 wt and from the agnA- strains was described previously [6]. The small RNA fraction from the rbdB- strain was prepared and sequenced in the same way. Around 5,3 M, 4,9 M and 6,6 M reads with sufficient quality for the AX2 wild type, the angA- strains and the rbdB- strain respectively were obtained. The processed and trimmed reads were mapped by the short read mapper *segmehl* [61] against the *D*. *discoideum* chromosomes (DDB0169550, DDB0215151, DDB0232428, DDB0232430, DDB0232432, DDB0237465, DDB0215018, DDB0220052, DDB0232429, DDB0232431, DDB0232433). The results were visualized and analyzed by the Integrative Genomics Viewer [62] or by the Integrated Genome Browser [63]. The data have been deposited in NCBI’s Gene Expression Omnibus (GEO) [64] and are available through GEO Series accession number GSE56111.

### Western Blot analysis

Western Blot analysis to verify gene expression of fusion proteins or to analyze Co-IP experiments were performed a described elsewhere [6].

### Co-immunoprecipitation

Co-IP experiments were performed as described elsewhere [65], except for the following modifications. 5 x 10^8^ cells were resuspended in 5 mL lysis buffer (10 mM Tris-Cl [pH 7.5], 150 mM NaCl, 0.5 mM EDTA, 0.5% NP-40, 25 mM MgCl_2_, 1 tablet of Roche proteinase inhibitor cocktail mini). After binding, the GFP-Trap beads (ChromoTek) were washed four times with a buffer containing 10 mM Tris-Cl (pH 7.5), 150 mM NaCl, 0.5 mM EDTA, 25 mM MgCl_2_. Bound protein was boiled off the beads in 80 μl Laemmli buffer. Aliquots were taken from the intermediate steps (Input, Preclear) and compared to bound protein by SDS-PAGE and subsequent Western blotting.

### Fluorescent microscopy of fixed *D*. *discoideum* cells

Images were acquired on a Leica DMIRB inverted microscope with a DC350 camera and IM50 Acquisition software (Leica Microsystems, Wetzlar, Germany) or on a Leica DM 5500 with a DFC365 camera and MMAF acquisition software. Around 2 x 10^5^ cells were plated on a coverslip to settle down for 20 minutes. Cells were washed with phosphate buffer, fixed in 4% Formalin for 5 min at 22°C and then permeabilized for 5 min in ice-cold methanol. Alternatively, cells were treated 7 minutes with methanol, only. Afterwards, cells were stained with DAPI for 3 minutes (DAPI stock solution (1 mg/mL) was diluted 1:15.0000 in 1 x PBS) and washed two times with 1 x PBS. The coverslips were mounted on a slide with a drop of mounting medium (90% (vol/vol) glycerol, 20 mM Tris-HCl, and 1 g ml1 1,4-diazabicyclo[2.2.2]octane (pH 8.3)). Living cells were incubated in *Low Fluorescence Axenic* Medium (Formedium, Hunstanton, UK) and analyzed on the Leica DMIRB.

## Supporting Information

S1 Fig Validation of *rbdA* and *rbdB* gene deletions. Schematic representation of the rbdA (A, top) and rbdB (B, top) wild type alleles, the knockout constructs and the null alleles after successful homologous recombination and insertion of the BsR cassette flanked by Lox-P sites (flox). We transformed each knockout construct twice in the AX2 wild type strain and selected one clone each from the independent transformations. These were denominated rbdA- [2], rbdA- [3], rbdB- [3] and rbdB- [5]. The BsR cassette from rbdB- strains was removed (rox) by transient expression of the Cre-recombinase from plasmid pDEX RH NLS-Cre resulting in the strains rbd- [1] (derived from rbdB- [3]) and rbdB- [2] (derived from rbdB- [5]). We confirmed the gene deletions by PCR analysis (A and B, bottom) and, in addition, performed Southern Blot analysis to exclude multiple integrations. Binding positions of primers 1–4 that were used to validate the gene deletions are indicated. Primers bind outside the targeting fragments. P1 = BB116, P2 = BB117, P3 = BB118, P4 = BB119 (see S1 Table). A, bottom: The *rbdA* gene deletion was verified by PCR on genomic DNA using P1 and P2 yielding fragments of 2631 bp for the rbdA- strain and 1835 bp for the AX2 wt. B, bottom: *rbdB* deletion was identified by PCR on genomic DNA using the primer set P3 and P4. Expected fragment sizes are 2730 for rbdB- strains and 2092 bp for the AX2 wt. Removal of the BsR cassette was confirmed by the same PCR. After successful deletion, the PCR results in a shorter product of 1320 bp.
(TIF)

S2 Fig DrnB and RbdB localize independent of each other. We monitored localization of DrnB GFP in the rbdB- strain and localization of RbdB mRFP in the drnB- strain, respectively by fluorescence microscopy. Both tagged proteins are mostly found in nucleoli associated foci though RbdB mRFP may also be more diffusely distributed in the nucleoli of some cells. We thus conclude that proteins localize independent of each other. Scale bar represents 10 μm.
(TIF)

S3 Fig Control of IP experiments. Different control co-immunoprecipitations were performed. Samples (IN = input, Pre = preclear, SN = supernatant, E = elution) were analyzed by Western Blots. A: RbdB (Δ 504–612) GFP and HcpA 3xHA were expressed in the AX2 wild type background and co-immunoprecipitation by GFP tagged RbdB (Δ 504–612) was performed. HcpA 3xHA could not be precipitated. B: GFP HcpA and DrnB 3xHA were expressed and co-immunoprecipitation was performed. DrnB 3xHA could not be precipitated by GFP HcpA.
(TIF)

S4 Fig srtA [RNAi] but not abpA [RNAi] knockdown strains show elevated miRNA levels/verification of srtA knockdown. A: Ddi-miR-7097 expression was analyzed by Northern Blot in the AX2 wt and in srtA [RNAi 1] and srtA [RNAi 2] knockdown strains. 12 μg total RNA were loaded per lane. Mature ddi-miR-7079 was detected by the 32P labelled oligonucleotide DM217. As a loading control, the snoRNA DdR6 was detected. B: Ddi-miR-1176 miRNA expression was analyzed in the AX2 wt and in abpA [RNAi] knockdown strains [48]. We could not detect any significant differences by Northern Blot analysis. C: *left*: *srtA* knockdown efficiency was analyzed by qRT-PCR analysis using an amplicon [A] that binds upstream of the trigger-sequence (see scheme). In case of srtA [RNAi] two biological and three technical replicates were analyzed. For AX2, six technical replicates were analyzed. SrtA [RNAi] showed a ~40% reduced *srtA* mRNA level. *Right*: Northern Blot analysis to show the generation of srtA specific siRNAs in the respective knockdown strain using ^32^P labelled oligonucleotides DM200 and DM201.
(TIF)

S5 Fig Expression of RbdB GFP from high copy and low copy integrating plasmids. A: *rbdB* or *rbdB gfp* mRNA levels were determined by qRT-PCR in the indicated strains. Expression levels in the AX2 were compared with rbdB- strains that were transformed with pDbsr2a RbdB GFP [LC] or with pDneo2a RbdB GFP [HC]. Values are means from two independent RNA preparations out of one biological replicate. B: Western Blot to analyze RbdB GFP expression levels in rbdB- strains that were transformed with pDbsr2a RbdB GFP [LC] or with pDneo2a RbdB GFP [HC]. Four biological replicates each were analyzed. Fusion proteins were only detectable when expressed from high copy integrating plasmids. Note that RbdB GFP fusion proteins (109 kDa) migrate more slowly than expected.
(TIF)

S6 Fig RbdB has a C-terminal NLS. A: Schematic representation of RbdB and truncated protein versions. Identified NLS and NoLS sequences are indicated in RbdB and in RbdB (Δ504–612). All signal sequences are absent in RbdB (Δ504–733). B: Amino acid sequence of the predicted signal sequences that were identified by the cNLS Mapper and by NoD, respectively. C. schematic representation of reporter proteins and subcellular localization. The predicted signal sequences and the linker were fused C-terminal to GFP. In addition, the NLS2 signal sequence alone was fused to GFP, respectively. As a control, GFP alone was expressed from the extrachromosomal vector pDM317 that was used for cloning. The predicted NLS2 was sufficient to shift cytoplasmic GFP into the nucleus. The predicted NLS and the NoLS seqence also shifted GFP to the nucleus but we could not observe any accumulation in the nucleoli or in nucleoli associated foci.
(TIF)

S7 Fig Visualization of miRNA expression in the wild type and in the angA- and rbdB- strains. Normalized RNA-seq data were visualized using the IGB browser [63]. Screen shots were taken. On the X-axis genomic coordinates are shown. Read Counts are shown on the y-axis. As a comparison, miRNAs ddi-miR-1176 and ddi-miR-1177 were shown, too [9].
(PDF)

S8 Fig validation of identified miRNAs (canonical miRNAs). A: Predicted hairpin structures of new miRNA candidates (canonical ones) and of ddi-mir-7097 [8] by M-fold [66]. Red: miRNA-5p, green: miRNA-3p. We only indicated the second miRNA, if it could be identified by our RNAseq approach. This was for example true for the previously identified miRNA ddi-mir-7097 [8]. B: Northern Blot analysis of identified miRNAs-candidates. 12 μg RNA from AX2 and indicated mutant strains were separated on an 11% PAA gel and transferred to a nylon membrane. miRNAs were detected with ^32^P labelled oligonucleotides. MiRNA_can_D2 was only detectable in agnA- and rrpC- strains. The same was true for miRNA_can_D3, even though with low signals intensity. The previously detected miRNA ddi-mir-7097 [8] as well as the corresponding miRNA-3p, whose existence could be confirmed by Illumina-RNA-sequencing, were detectable in the AX2 wild type and in the expected mutant strains.
(TIF)

S1 Table Oligonucleotides used in this study. (DOCX)

S2 Table Specification of knockout plasmids. The position of the knockout arms is given relative to the start codon of the respective gene. In addition, the number of deleted base pairs is annotated.
(DOCX)

S3 Table Absolute and relative read counts of identified miRNAs. Absolute read counts are given for the denoted genomic positions. Relative read counts were calculated as follows: absolute read counts were divided by the total number of aligned reads and then normalized to the AX2.
(XLSX)

S4 Table Identified miRNAs. Genome positions of the identified miRNAs are shown. The coordinates may deviate by a few nucleotides. Plus and minus indicate the respective strand.
(DOCX)
