# Supplementary figures and images for: Analysis of the Microprocessor in Dictyostelium: The Role of RbdB, a dsRNA Binding Protein
Source: PLoS Genet. 2016 Jun 6;12(6):e1006057. doi: 10.1371/journal.pgen.1006057 (PMC4894637; doi:10.1371/journal.pgen.1006057)

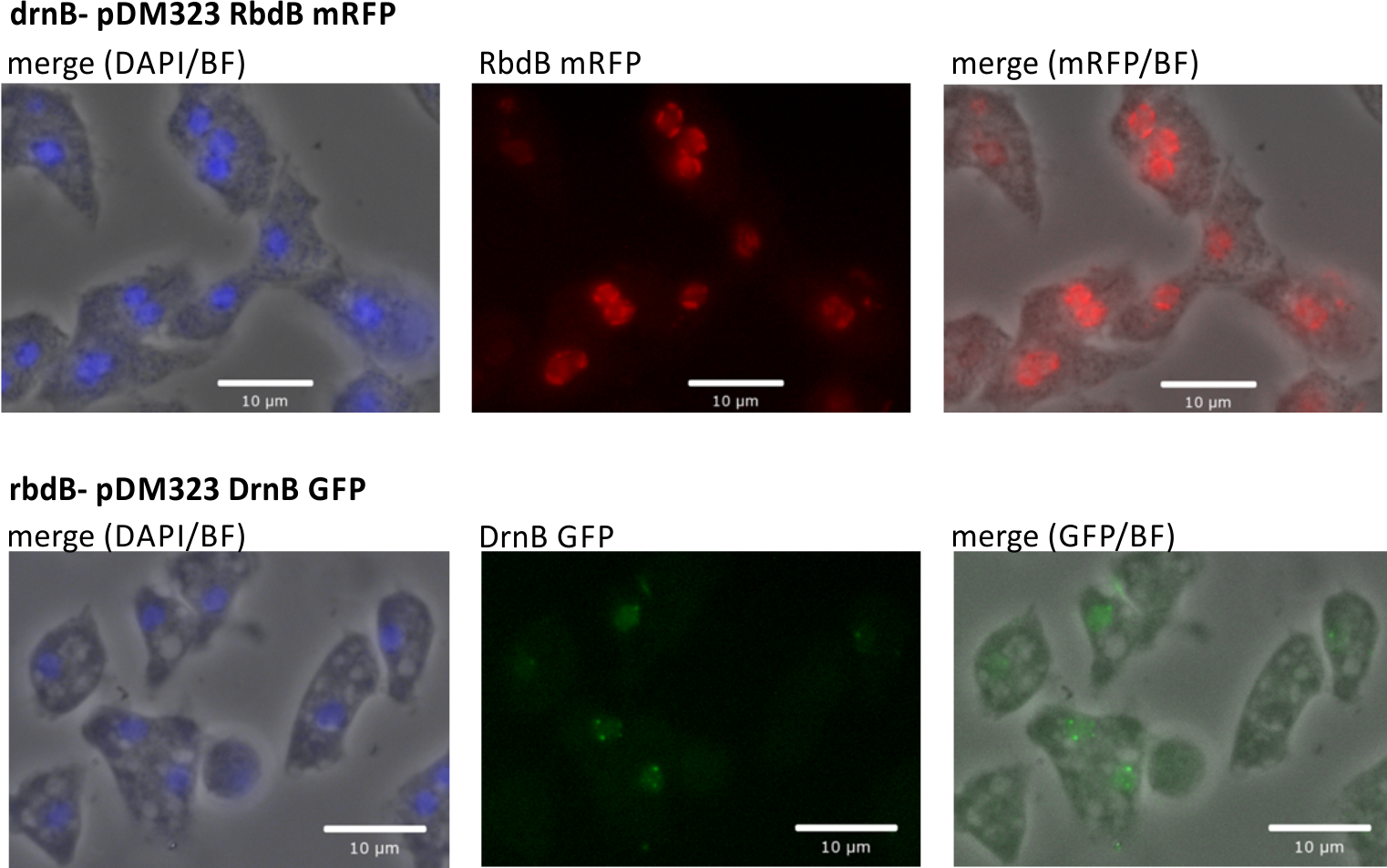

Supplement: S2 Fig — We monitored localization of DrnB GFP in the rbdB- strain and localization of RbdB mRFP in the drnB- strain, respectively by fluorescence microscopy. Both tagged proteins are mostly found in nucleoli associated foci though RbdB mRFP may also be more diffusely distributed in the nucleoli of some cells. We thus conclude that proteins localize independent of each other. Scale bar represents 10 μm. (TIF) [file pgen.1006057.s002.tif]

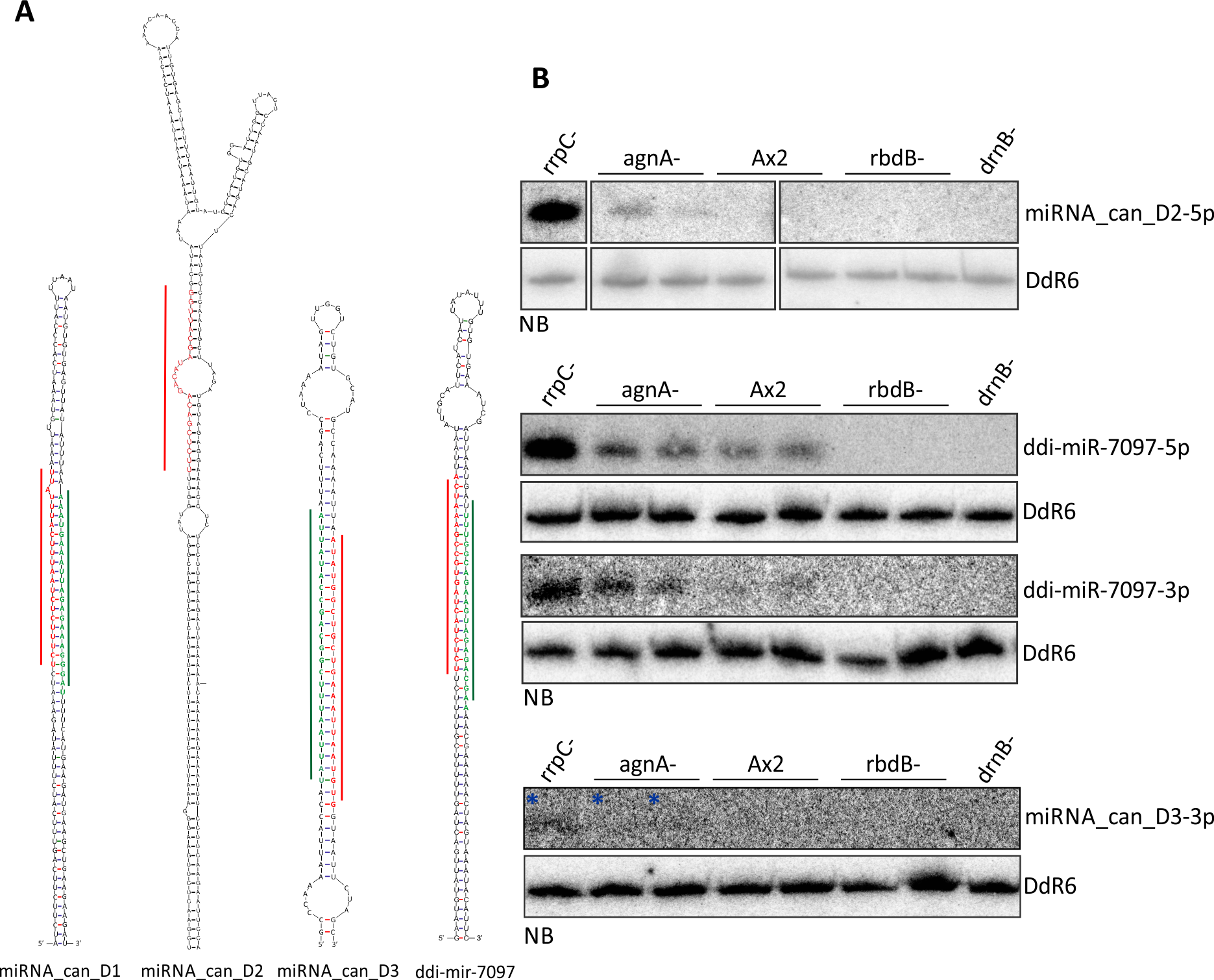

Supplement: S8 Fig — A: Predicted hairpin structures of new miRNA candidates (canonical ones) and of ddi-mir-7097 [8] by M-fold [66]. Red: miRNA-5p, green: miRNA-3p. We only indicated the second miRNA, if it could be identified by our RNAseq approach. This was for example true for the previously identified miRNA ddi-mir-7097 [8]. B: Northern Blot analysis of identified miRNAs-candidates. 12 μg RNA from AX2 and indicated mutant strains were separated on an 11% PAA gel and transferred to a nylon membrane. miRNAs were detected with 32P labelled oligonucleotides. MiRNA_can_D2 was only detectable in agnA- and rrpC- strains. The same was true for miRNA_can_D3, even though with low signals intensity. The previously detected miRNA ddi-mir-7097 [8] as well as the corresponding miRNA-3p, whose existence could be confirmed by Illumina-RNA-sequencing, were detectable in the AX2 wild type and in the expected mutant strains. (TIF) [file pgen.1006057.s008.tif]
