# Supplementary material for: Analysis of the Microprocessor in Dictyostelium: The Role of RbdB, a dsRNA Binding Protein
Source: PLoS Genet. 2016 Jun 6;12(6):e1006057. doi: 10.1371/journal.pgen.1006057 (PMC4894637; doi:10.1371/journal.pgen.1006057)

miRNA D1-5p_miRNA D1-3p

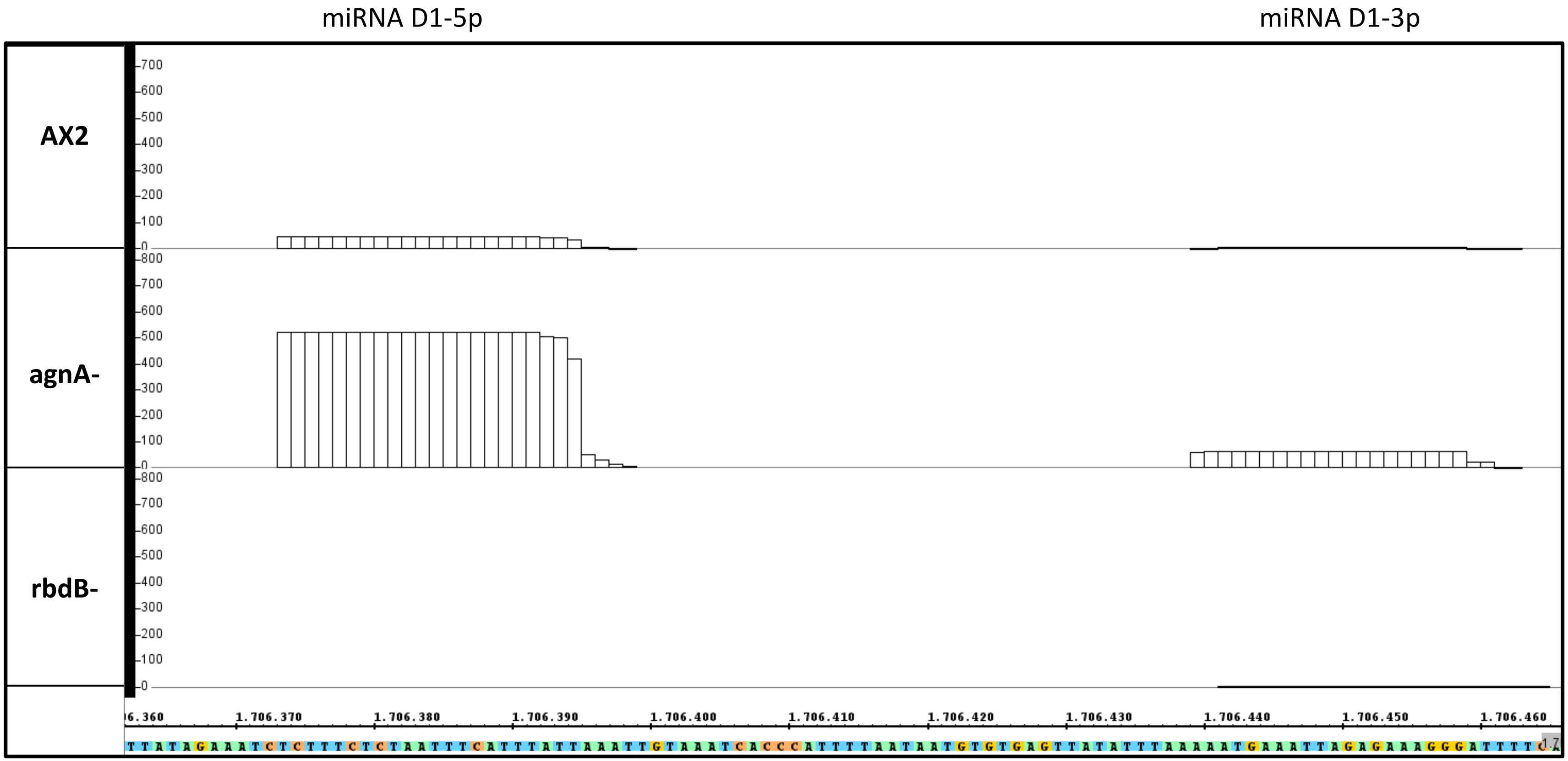

miRNA D2-5p_miRNA D2-3p

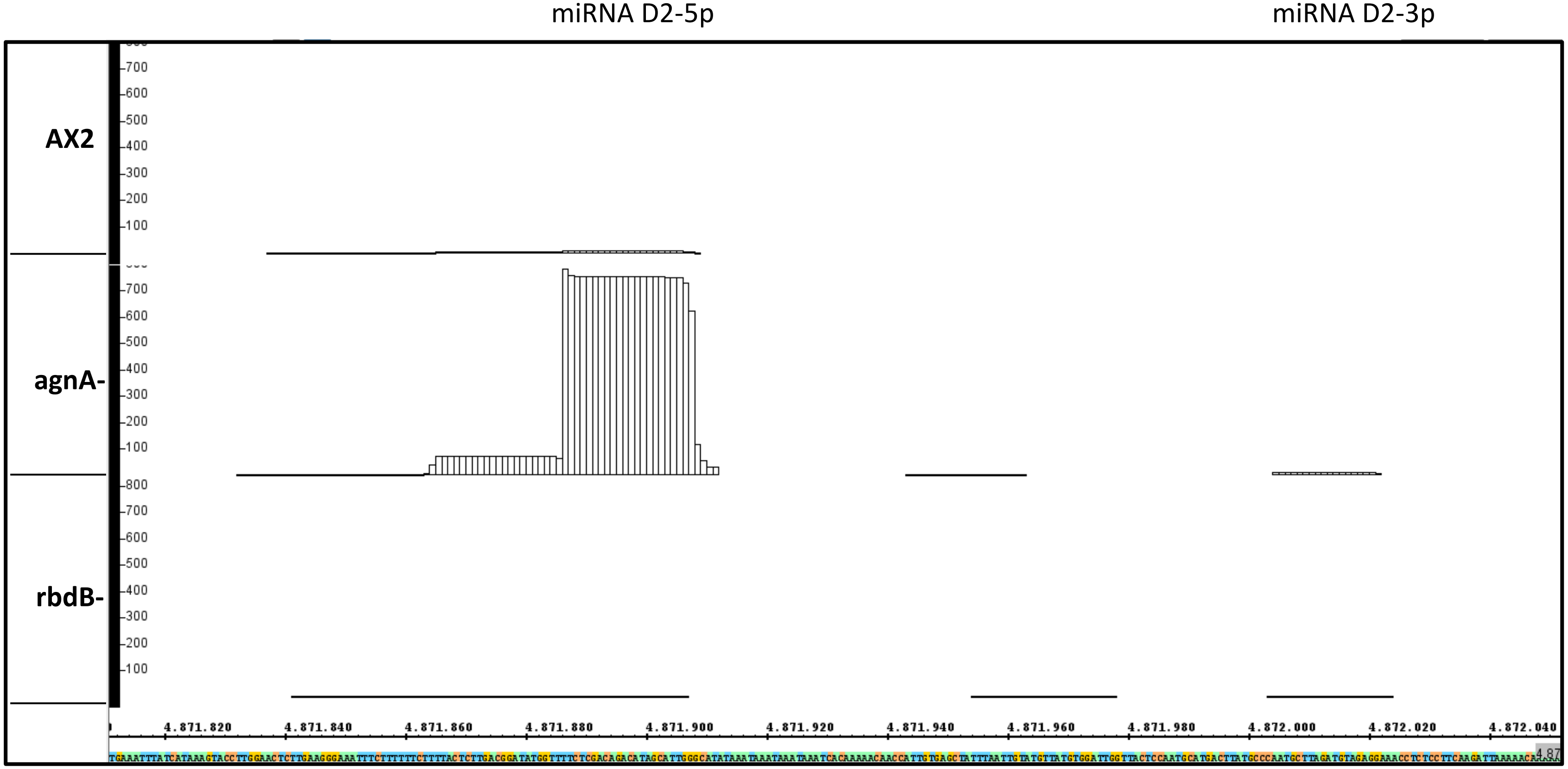

miRNA D3-5p_miRNA D2-3p

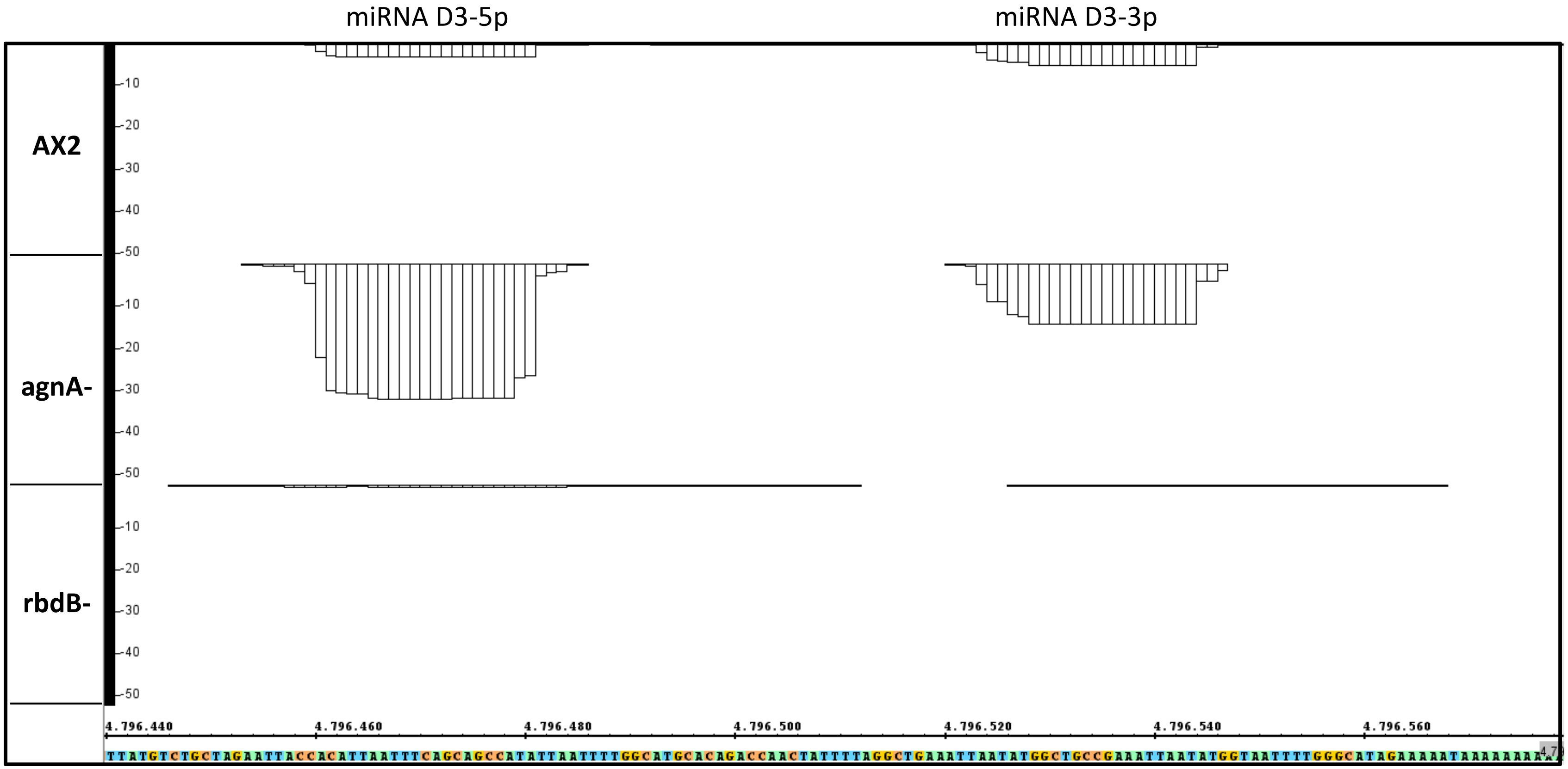

small RNA D4

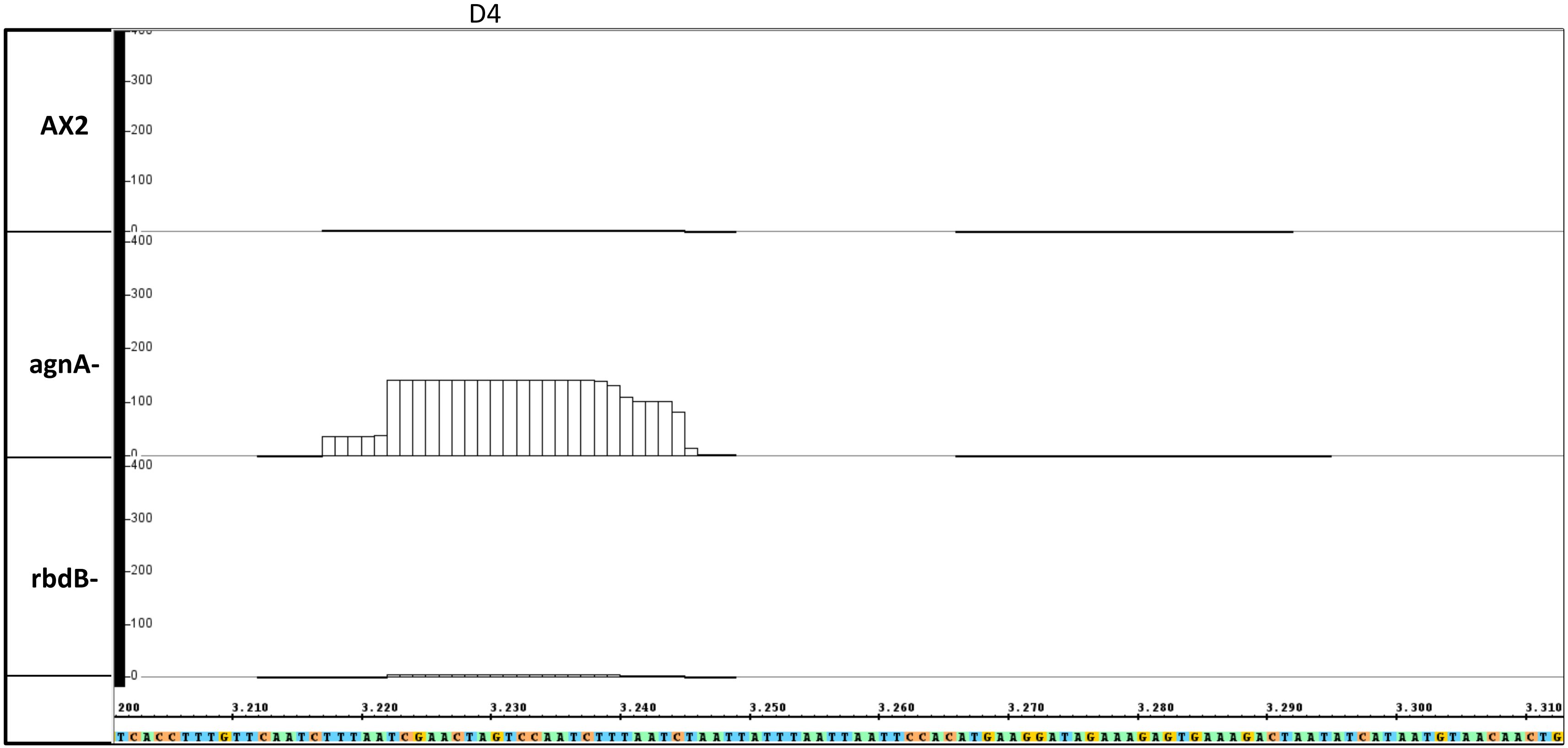
D4
AX2
agnA-
rbdB-
400
300
200
100
0
200
3.210
3.220
3.230
3.240
3.250
3.260
3.270
3.280
3.290
3.300
3.310

ddi-miR-7097_ddi-miR-7097*

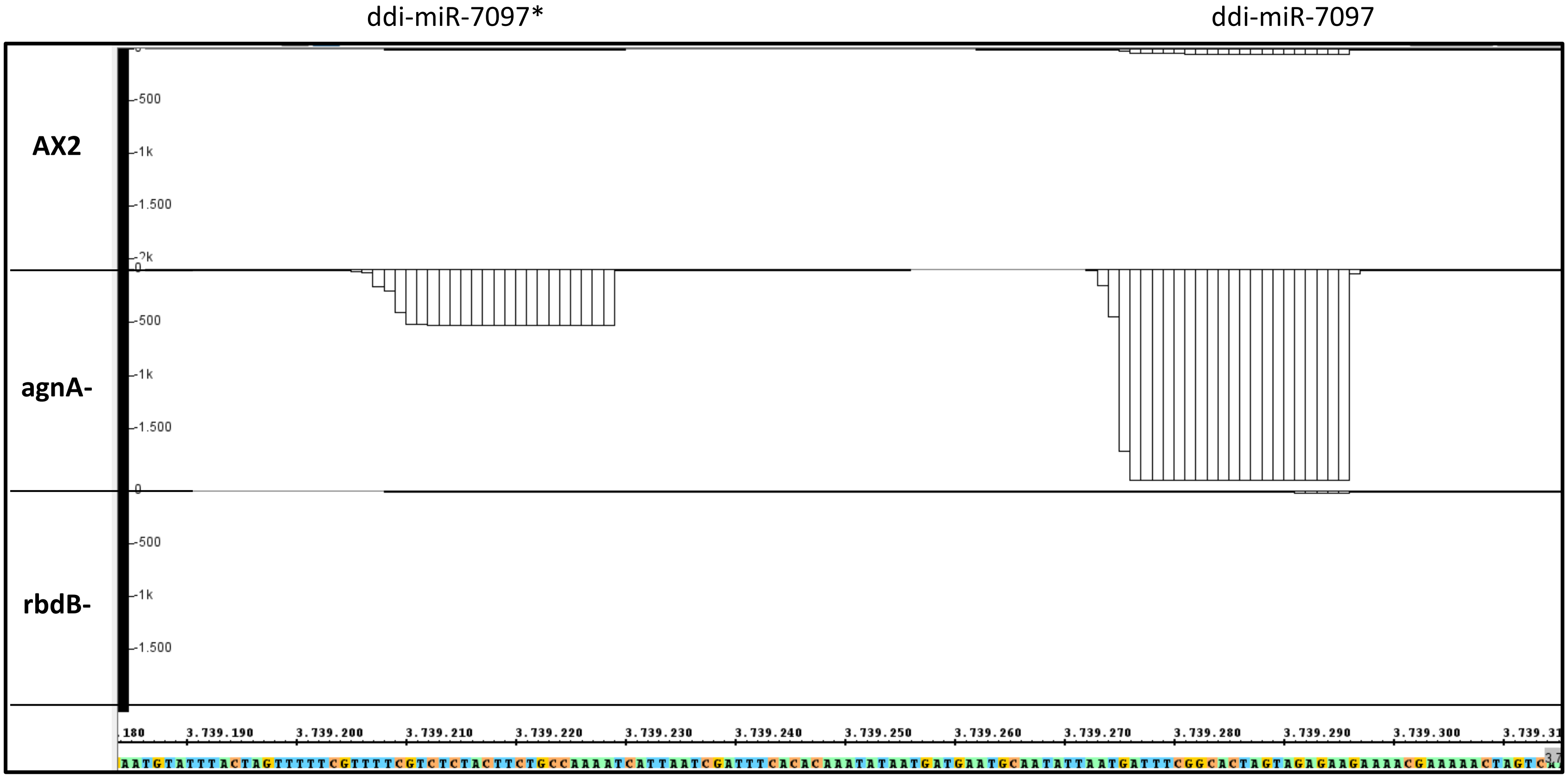

ddi-miR-1176_ddi-miR-1176*

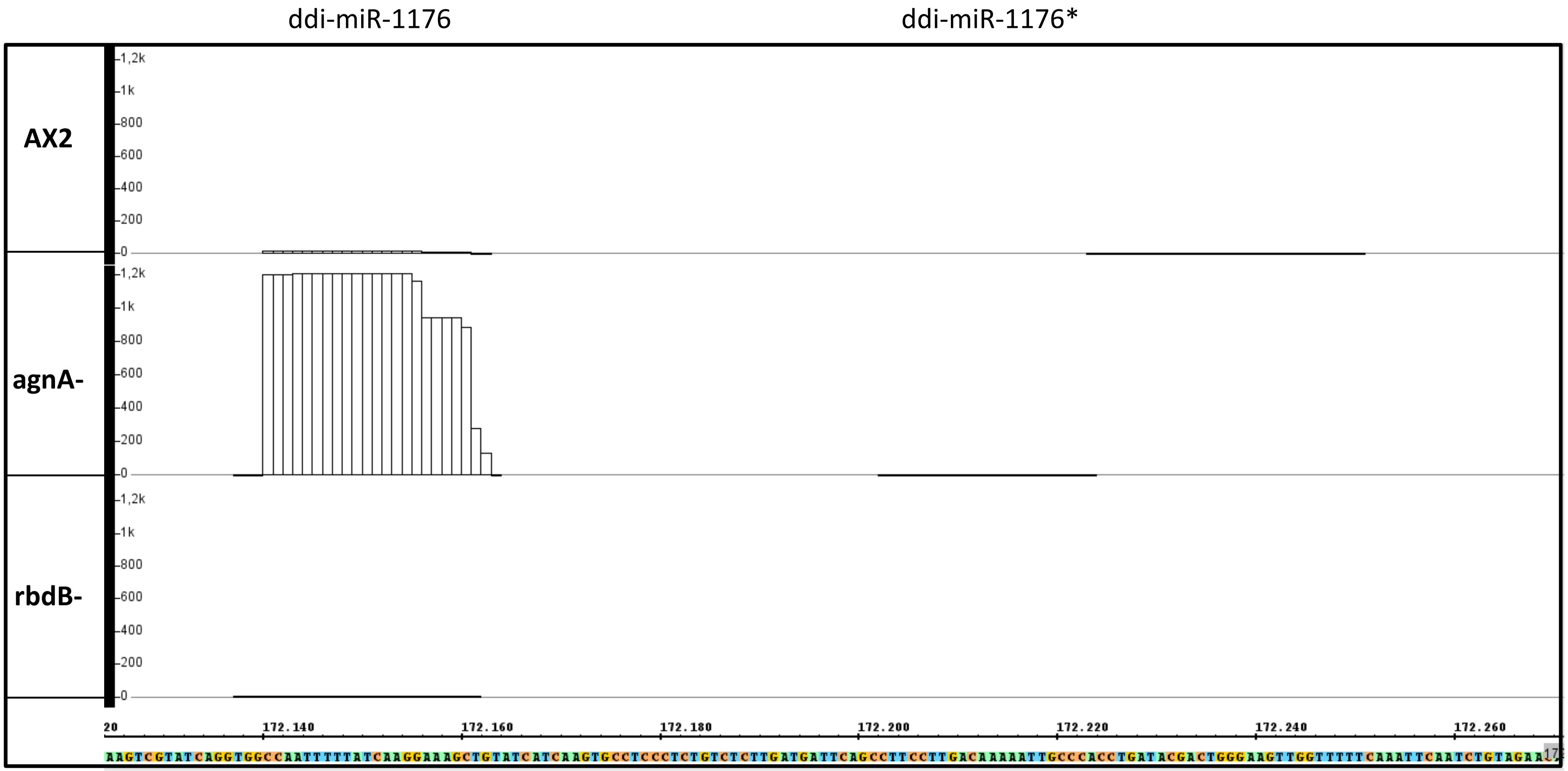

ddi-miR-1177_ddi-miR-1177*

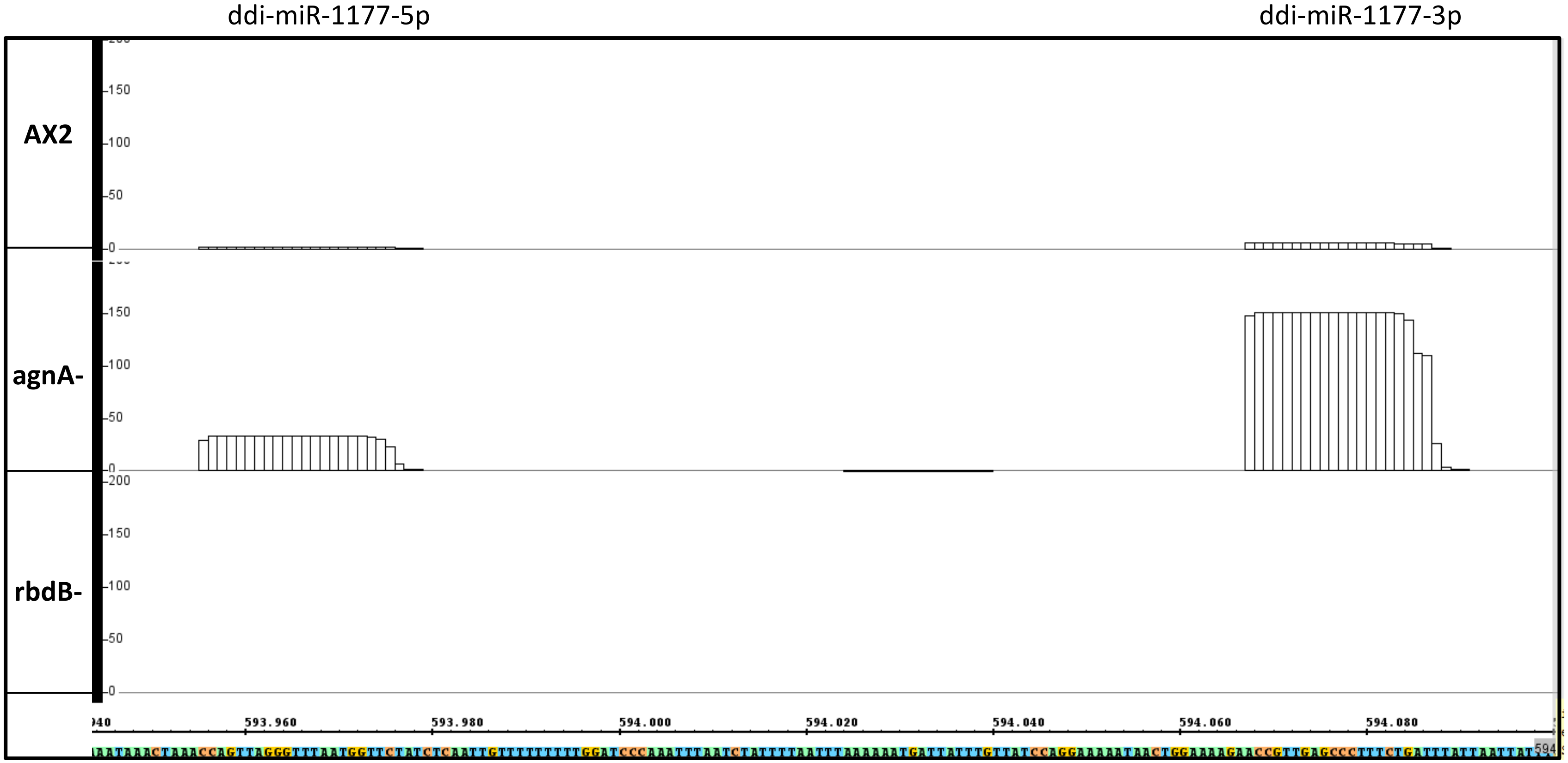

Supplement: S7 Fig — Normalized RNA-seq data were visualized using the IGB browser [63]. Screen shots were taken. On the X-axis genomic coordinates are shown. Read Counts are shown on the y-axis. As a comparison, miRNAs ddi-miR-1176 and ddi-miR-1177 were shown, too [9]. (PDF) [file pgen.1006057.s007.pdf]
