# Supplementary material for: Analysis of the Microprocessor in Dictyostelium: The Role of RbdB, a dsRNA Binding Protein
Source: PLoS Genet. 2016 Jun 6;12(6):e1006057. doi: 10.1371/journal.pgen.1006057 (PMC4894637; doi:10.1371/journal.pgen.1006057)
Supplement: S1 Table — (DOCX) [file pgen.1006057.s009.docx]

**Table S1: Oligonucleotides**

| **name** | **sequence** | **application** |
| --- | --- | --- |
| BB100  BB101 | Agcgcgtctccaatgctgcagccaaatcagataatcctggc6  Agcgcgtctccgttggcggtgctggtggagcca6 | left arm pKOSG *rbdA* ko plasmid |
| BB102  BB103 | Agcgcgtctcccttcgacaaagaaacaagcccaagctgc6  Agcgcgtctcctcccctgcaggattcttcaagtttaatttgaagttg6 | right arm pKOSG *rbdA* ko plasmid |
| BB104  BB105 | agcgcgtctccaatgctgcaggagataatcgagatgattggtaactc6  Agcgcgtctccgttggtaaaacttgaaatctttgaaaattgg6 | left arm pKOSG *rbdB* ko plasmid |
| BB106  BB107 | agcgcgtctcccttcgtctatagtagtagtaaatggaaaac6  Agcgcgtctcctcccctgcagcgttgttcgagatggataagtttg6 | right arm pKOSG *rbdB* ko plasmid |
| BB116  BB117 | CATCTTTTATTGCTGGAGTATACC6  CAATGTCTCCATTCTCTTTGTTAATTC6 | screen of *rbdA* mutants |
| BB118  BB119 | ATTATTTGAATCCACCCCAAACCC6  GTTTTTGTAATTGTTGTGTATGTCCAG6 | screen of *rbdB* mutants |
| DM033  DM034 | CTGCAGAAATGTCAAATAAATCTCATACATCAGAAAAGG6  CTGCAGCTTTTTTTGAAATTAAATTTTGTATTGG6 | amplification of *rbdB* gene, PstI flanked |
| DM066  DM053 | tgatcaaaaatgtcaaataaatctcatacatcag6  actagttttttttgaaattaaattttgtattgg6 | amplification of *rbdB* gene, BclI/SpeI flanked |
| DM196  DM197 | GGATCCAAAATGGAACAAGAAGAAGCAGTTGATGG  GGTACCACATTTTCTAGTGGTTTCACC | amplification of *serrate*, 1^st^ part BamHI/KpnI flanked |
| DM198  DM199 | GGTACCATTCATTTAAGAAGAACACAGG  ACTAGTTAAACTCTTTTTATTTTGATATTCTTTAAGC | amplification of *serrate*, 2^nd^ part, KpnI/SpeI flanked |
| DM167  DM172 | TAAATTAGATCTAAAATGTTGAAAAGAAAGTTGGAAGAATCTCCAACAG  ACTAGTAATAGGAGAGGGGCTTGTAGGTGGG | amplification of the NLS-NoLS [RbdB] sequence |
| DM167  DM168 | TAAATTAGATCTAAAATGTTGAAAAGAAAGTTGGAAGAATCTCCAACAG  TAAATTACTAGTAATCTTTTGTAGTTTGCTCATCAATGTTGGAGATTCTG | Recursive PCR: generation of the NLS [RbdB] sequence |
| DM058  DM059 | CACCAATGGTCAAACAAAATGTTTTTGCTG1  GATCAGAGAAGAATCAAAATATTGGGTG1 | RT-PCR to detect pri-ddi-miR-1176 |
| #3169  #3170 | ACTAGTACATATCCATACGATGTTCCAGATTATGCAGGAGGATACCCTTATGATGTACTGACTACGCA1  TCTAGACCCACCTGCATAATCTGGAACATCGTATGGATAACCACCTGCGTAGTCAGGTACATCATAAGG1 | recursive PCR, 3xHA tag, SpeI/XbaI flanked |
| FZ005  FZ008 | AGATCTAAAATGTCAAATAAATCTCATTTATCTGAAAAGG1  ACTAGTATCAGTTTCAGAGAATTTTAAATCATC1 | amplification of drnB, BglII/SpeI flanked |
| DM066  DM065 | tgatcaaaaatgtcaaataaatctcatacatcag  AGATCTCTGAGTATCAACTCCTAAAACAATAGTATTGG | construction of N-terminal part of rbdB Δ504-612 BclI/BglII |
| DM066  JB002 | tgatcaaaaatgtcaaataaatctcatacatcag  ACTAGTCTGAGTATCAACTCCTAAAACAATAGTATTG | amplification of rbdB Δ504-733 BclI/SpeI flanked |
| #2600 | GCTTTCCTTGATAAAAATTGG | α-1176, [[9](#_ENREF_9)] |
| #2602 | GAACCATTAAACCCTAACTGG | α-1177, [[9](#_ENREF_9)] |
| #2654 | GGCCAACAATTTTCTCAGCAAGAC | α sno6 (DdR6) |
| DM122 | AAATCCCTTTCTCTAATTTCATTT | α miRNA_can_D1-3p |
| #2952 | ATTAAAGATTGGACTAGTTCGA | α miRNA-like_D4 |
| DM123 | CCAATGCTATGTCTGTCGAGAA | α miRNA _can_D2 |
| DM227 | CCACATTAATTTCAGCAGCCATA | α miRNA_ can_D3 |
| #1828  #1829 | AAATATCGTCATGTTTTTGCAGCACAAC  AATAATGGAACTGATGTGGTTTTACCTGAA | RT-PCR, α *corA* |
| DM058  DM059 | CACCAATGGTCAAACAAAATGTTTTTGCTG1  GATCAGAGAAGAATCAAAATATTGGGTG1 | RT-PCR, α pri-ddi-miR-1176 |
| DM082  DM083 | TGGAATGGTTACAAATCTTATAAATAG1  AGTAACCATAATGTAGTCATCAAATAA1 | RT-PCR, α ddi-miR-1177 |
| DM167  DM172 | TAAATTAGATCTAAAATGTTGAAAAGAAAGTTGGAAGAATCTCCAACAG  ACTAGTAATAGGAGAGGGGCTTGTAGGTGGG | amplification of NLS-NoLS signal sequences from the *rbdB* gene |
| DM208  DM209 | AGATCTAAAATGTTGAAAAGAAAGTTGGAAGAATCTCCAACATTGATGAGCAAAC  ACTAGTCTCTTCTTTTTCAAATTGAATCTTTTGTAGTTTGCTCATCAATGTTGGA | amplification of the *rbdB* NLS2 sequence by recursive PCR |
| DM200  DM201 | AGATCTTATAAACAGTTTTTAGATTATC.  ACTAGTGTTACCCAACCTAAACGATAAAAG | amplification of srtA RNAi trigger |
| #1923  #1924 | GCCAGAAATGCTTTGAAAATGACAC  GAGTGGTTTGCCAATTTCTTTTCCT | qRT-PCR amplicon (cinD) |
| #3050  #3051 | AGTTCGTCCGATAGTGATAGTGA  ACCACCATCCCAATCATCTCT | qRT-PCR amplicon (srtA) |
