# Supplementary material for: Analysis of the Microprocessor in Dictyostelium: The Role of RbdB, a dsRNA Binding Protein
Source: PLoS Genet. 2016 Jun 6;12(6):e1006057. doi: 10.1371/journal.pgen.1006057 (PMC4894637; doi:10.1371/journal.pgen.1006057)
Supplement: S2 Table — The position of the knockout arms is given relative to the start codon of the respective gene. In addition, the number of deleted base pairs is annotated. (DOCX) [file pgen.1006057.s010.docx]

**Table S2: Specification of knockout plasmids**

| **gene-knockout** | **plasmid** | **left arm** | **right arm** | **deletion of** |
| --- | --- | --- | --- | --- |
| *rbdA* | pKOSG rbdA - | -999 to -552 | +157 to +749 | 709 bp |
| *rbdB* | pKOSG rbdB - | -883 to -503 | +362 to +1.106 | 865 bp |
