# Supplementary material for: Analysis of the Microprocessor in Dictyostelium: The Role of RbdB, a dsRNA Binding Protein
Source: PLoS Genet. 2016 Jun 6;12(6):e1006057. doi: 10.1371/journal.pgen.1006057 (PMC4894637; doi:10.1371/journal.pgen.1006057)
Supplement: S4 Table — Genome positions of the identified miRNAs are shown. The coordinates may deviate by a few nucleotides. Plus and minus indicate the respective strand. (DOCX) [file pgen.1006057.s012.docx]

| ***miRNA** | **Chromosome** | **position** | **strand** |
| --- | --- | --- | --- |
| ***miRNA** |  |  |  |
| **canonical miRNAs** | | |  |
| miRNA_can_D1 | 4 | 1706374..1706395 | plus |
| miRNA_can_D1* | 4 | 1706440..1706461 | plus |
| miRNA_can_D2 | 5 | 4871887..4871908 | plus |
| miRNA_can_D2* | 5 | 4872005..4872026 | plus |
| ddi-mir-7097 | 3 | 3739275..3739295 | minus |
| ddi-mir-7097* | 3 | 3739208..3739228 | minus |
| miRNA_can_D3 | 5 | 4796459..4796481 | minus |
| miRNA_can_D3* | 5 | 4796524..4796544 | minus |
| miRNA_can_D3 | 5 | 425213..425235 | plus |
| miRNA_can_D3* | 5 | 425150..425170 | plus |
| miRNA_can_D3 | 3 | 4529863..4529841 | minus |
| miRNA_can_D3* | 3 | 4529926..4529906 | minus |
| miRNA_can_D3 | 2 | 1631003..1630981 | minus |
| miRNA_can_D3* | 2 | 1631066..1631046 | minus |
| miRNA_can_D3 | 2 | 2783137..2783116 | minus |
| miRNA_can_D3* | 2 | 2783200..2783180 | minus |
| miRNA_can_D3 | 2 | 3248649..3248671 | plus |
| miRNA_can_D3* | 2 | 3248586..3248606 | plus |
| **non-canonical miRNA** | | |  |
| miRNA_non_can_D4 | 6 | 3669..3690 | plus |
| miRNA_non_can_D4* | nd | - |  |
| miRNA_non_can_D4 | 4 | 3223..3244 | plus |
| miRNA_non_can_D4* | nd | - | - |
| miRNA_non_can_D4 | 3 | 10372..10351 | minus |
| miRNA_non_can_D4* | nd | - | - |
| miRNA_non_can_D4 | 2 | 3773252..3773232 | minus |
| miRNA_non_can_D4* | nd |  |  |

**Table S4**
